# Recommendations for hemodynamic monitoring for critically ill children—expert consensus statement issued by the cardiovascular dynamics section of the European Society of Paediatric and Neonatal Intensive Care (ESPNIC)

**DOI:** 10.1186/s13054-020-03326-2

**Published:** 2020-10-22

**Authors:** Yogen Singh, Javier Urbano Villaescusa, Eduardo M. da Cruz, Shane M. Tibby, Gabriella Bottari, Rohit Saxena, Marga Guillén, Jesus Lopez Herce, Matteo Di Nardo, Corrado Cecchetti, Joe Brierley, Willem de Boode, Joris Lemson

**Affiliations:** 1 Department of Pediatrics - Neonatology and Pediatric Cardiology, Cambridge University Hospitals and University of Cambridge School of Clinical Medicine, Biomedical Campus, Hills Road, Cambridge, CB2 0QQ UK; 2 Department of Pediatric Intensive Care, Gregorio Marañón Hospital University Hospital, Madrid, Spain; 3 Department of Pediatrics, Children’s Hospital Colorado, Section of Cardiac Intensive Care, The Heart Institute, Pittsburgh, USA; 4 Department of Pediatric Intensive Care, Evelina London Children’s Hospital, London, UK; 5 Department of Pediatric Intensive Care, Ospedale Pediatrico Bambino Gesù-IRCC, Rome, Italy; 6 Department of Pediatric and Cardiac Intensive Care, Great Ormond Street Hospital for Children and UCL Institute for Child Health, London, UK; 7 Department of Pediatric Intensive Care, The Newcastle Upon Tyne Hospitals NHS Foundation Trust, Newcastle, UK; 8 Department of Neonatology, Radboud University Medical Center, Radboud Institute for Health Sciences, Amalia Children’s Hospital, Nijmegen, The Netherlands; 9 Department of Intensive Care Medicine, Radboud University Medical center, Radboud Institute for Health Sciences, Nijmegen, The Netherlands

**Keywords:** Hemodynamic monitoring (HD), Paediatric intensive care unit (PICU), Children, Cardiovascular instability, Recommendations

## Abstract

**Background:**

Cardiovascular instability is common in critically ill children. There is a scarcity of published high-quality studies to develop meaningful evidence-based hemodynamic monitoring guidelines and hence, with the exception of management of shock, currently there are no published guidelines for hemodynamic monitoring in children. The European Society of Paediatric and Neonatal Intensive Care (ESPNIC) Cardiovascular Dynamics section aimed to provide expert consensus recommendations on hemodynamic monitoring in critically ill children.

**Methods:**

Creation of a panel of experts in cardiovascular hemodynamic assessment and hemodynamic monitoring and review of relevant literature—a literature search was performed, and recommendations were developed through discussions managed following a Quaker-based consensus technique and evaluating appropriateness using a modified blind RAND/UCLA voting method. The AGREE statement was followed to prepare this document.

**Results:**

Of 100 suggested recommendations across 12 subgroups concerning hemodynamic monitoring in critically ill children, 72 reached “strong agreement,” 20 “weak agreement,” and 2 had “no agreement.” Six statements were considered as redundant after rephrasing of statements following the first round of voting. The agreed 72 recommendations were then coalesced into 36 detailing four key areas of hemodynamic monitoring in the main manuscript. Due to a lack of published evidence to develop evidence-based guidelines, most of the recommendations are based upon expert consensus.

**Conclusions:**

These expert consensus-based recommendations may be used to guide clinical practice for hemodynamic monitoring in critically ill children, and they may serve as a basis for highlighting gaps in the knowledge base to guide further research in hemodynamic monitoring.

## Introduction

Circulatory shock is defined as a “life-threatening generalized the maldistribution of blood flow resulting in failure to deliver and/or utilize the adequate amount of oxygen, leading to tissue dysoxia” [1]. Cardiovascular instability with or without shock is common in children admitted to pediatric intensive care units. Over half of the children with hemodynamic instability in intensive care units have multiple-organ dysfunction and sepsis remains the leading cause [2]. Similarly, a multi-center international study (Sepsis Prevalence, Outcomes, and Therapies; SPROUT) reported that over two thirds of children with sepsis had multiorgan dysfunction, which was associated with a very high mortality. These data are similar to what has been described in the adult population [3]. However, there remains a paucity of data regarding the epidemiology of circulatory derangement in children and how best hemodynamic status can be evaluated or monitored in the pediatric intensive care units.

Multiple studies have established that early recognition and treatment of pediatric circulatory insufficiency or shock are crucial to improve survival. However, the optimal way to resuscitate children with circulatory failure is controversial. The exact order and quantity of fluids or vasoactive drug administration in the critically ill child with shock is supported by little evidence, although there are several consensus statements [4, 5].

Overzealous fluid resuscitation is detrimental to some children [6, 7]. There is some consensus regarding the first-line treatment in patients with shock or significant hemodynamic instability, but debate remains concerning how much fluid should be administered, which type of vasoactive drug should be used, how to assess the hemodynamic changes, and ultimately what hemodynamic clinical goal should be targeted to guide optimal treatment.

Ideal hemodynamic monitoring should accurately determine the severity of circulatory derangement and illustrate the underlying pathophysiologic mechanism to enable the clinician to choose the most appropriate treatment and to guide the therapy [7, 8]. Furthermore, hemodynamic monitoring can provide useful information about the circulatory condition in almost all types of life-threatening shock. This can be hemodynamic instability as a result of major surgery, cardiac failure, trauma, sepsis, or other causes of shock in children. The current guidelines mostly focus on early recognition and treatment but do not specify the type of monitoring technology that can or should be used under various circumstances. There are still no published evidence-based or even consensus guidelines specifically for hemodynamic monitoring in neonates and children. However, there is a wide range of devices and techniques available to evaluate the hemodynamic status and an increasing number of these methods have become available for use in children. In the annual meeting of the European Society of Paediatric and Neonatal Intensive Care (ESPNIC) in October 2016, members of the Cardiovascular Dynamics Section were tasked to develop evidence-based guidelines if at all possible, or an expert consensus statement on the hemodynamic monitoring specifically for use in children.

## Methods

A steering committee (SC) of three lead authors, one pediatric intensivist/anesthesiologist (JL), one neonatologist/pediatric cardiologist (YS), and one pediatric intensivist (JU), identified nine expert panel members who significantly contributed with publications in hemodynamic monitoring or cardiovascular status assessment in children in the last 10 years, similarly to what had been done with previous ESPNIC guidelines [9–11]. Further, the three selection criteria for inclusion as panel member were (1) must be clinicians working in a pediatric or neonatal intensive care, (2) needed to have experience with some form of HD monitoring, and (3) should have published in peer-reviewed journals concerning the topic. Panelists’ selection was performed prior to the literature search, and for logistic reasons, the number of participants was limited to a maximum of 12. All invited experts agreed to participate.

The working group had face-to-face meetings during the ESPNIC conference in Lisbon 2017 and the European Academy of Paediatric Societies (EAPS) conference in Paris in 2018 and unanimously agreed to provide recommendations from full-term infants over 37 weeks of gestation and over 4 weeks of postnatal age (lower limit) to 18 years old (upper limit), in order not to overlap with preterm-neonatal and adult guidelines. The panel subdivided hemodynamic monitoring into 12 subgroups: arterial blood pressure, central venous pressure, pulmonary artery catheter, cardiac output, transpulmonary thermodilution, central venous oxygen saturation measurement, lactate levels, clinical signs, near-infrared spectroscopy, fluid responsiveness, microcirculation, and role of ultrasonography.

Panel members were assigned in pairs to one of the subgroups, and each subgroup was coordinated by one of the steering committee members. The tasks of each subgroup consisted of performing a thorough review of the literature, writing a short description of the parameter/method, the technical background and physiological basis, writing a short overview of the reliability of the method if applicable, establishing, when possible, normal or target values, estimating the clinical value of the method, or parameter in relation to the patient categories mentioned. Because of the hemodynamic knowledgeable background of the panel members, these documents served as an overview to provide the entire panel with recent collective knowledge. The documents were not intended as a structured systemic review of the specific technology or method. Also, given that there is only low-quality data available in many aspects of hemodynamic monitoring in children, and that the focus of this work was to reach a consensus within the panel of experts, the working group decided not to use the GRADE system to evaluate the literature [12]. The definition of hemodynamic instability was not ill-defined but intended as a clinical description reflecting the child in need for fluid resuscitation or vasoactive drugs.

Finally, 3 types of recommendations were formulated as follows: (1) recommendations considering the reliability of methods, but only if applicable; (2) recommendations considering normal or target values, but only if applicable; and (3) recommendations considering clinical use in relation to specific patient groups.

The setup and some proposed recommendations were discussed in a face-to-face meeting during the European Academy of Pediatric Societies (EAPS) in Paris, France (October 2018). In May 2019, an anonymous electronic voting system (Survey Monkey®, San Mateo, USA) was used to vote on all recommendations by each panel member including the SC. Each panel member was given access to all the work from other subgroups with text, results, and full-text publications in order to vote with all available evidence. Panel members scored all the recommendations individually from 1 (complete disagreement) to 9 (complete agreement). The median score was calculated after eliminating one lowest and one highest value. Recommendations were labeled “strong agreement” (median 7–9 and with no individual score < 7), “equipoise” (median 4–6), or “disagreement” (median 1–3).

All recommendations that reached “strong agreement” in the first voting round according to the above scoring system acquired the strong agreement label. All recommendations that reached “equipoise” or “disagreement” in the first voting round were discussed and rephrased in the panel meeting during the ESPNIC conference in Salzburg, Austria (June 2019). The revised recommendations underwent the same voting process as in the first round. The revised recommendations retaining “strong agreement” after the second electronic voting were labeled as “weak agreement.” All other revised recommendations were classified as “no agreement.” Guidelines have been prepared according to the international Appraisal of Guidelines, Research and Evaluation (AGREE) [13].

## Results

**Fig. 1:**
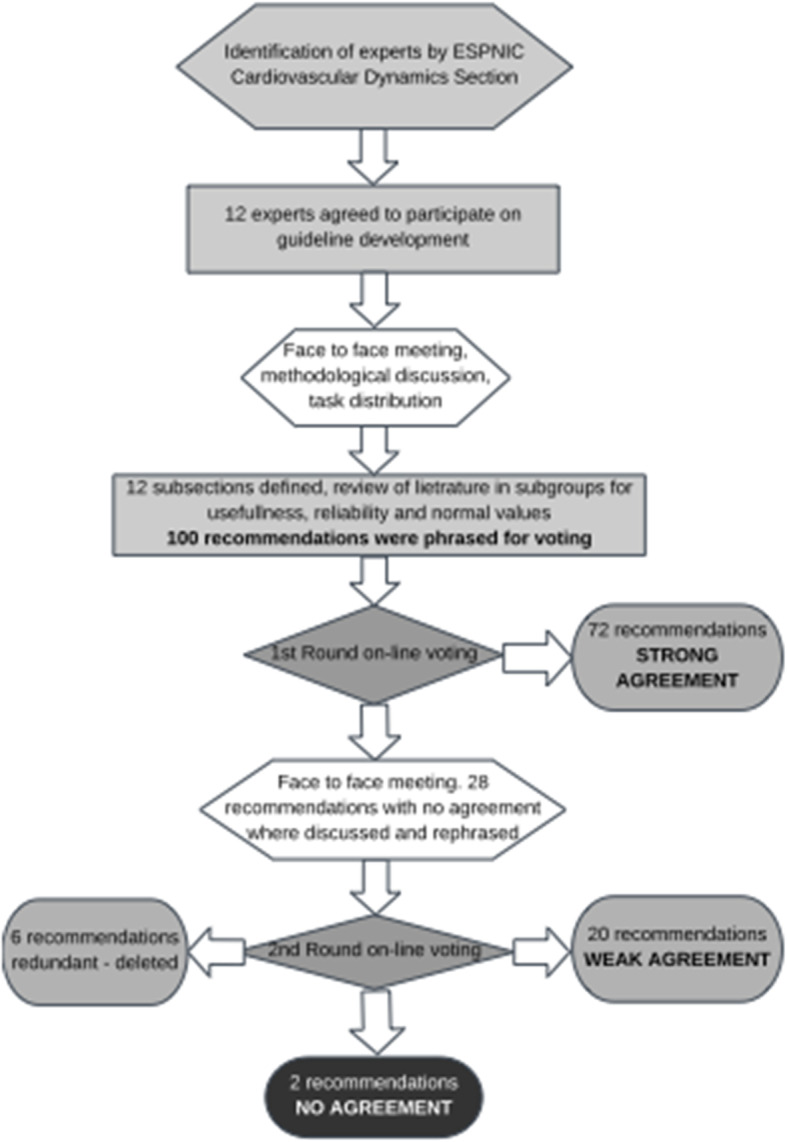
Flow chart of the methodology used in consensus development

One hundred recommendations were drafted and voted by the panel for the level of agreement. Seventy-two recommendations reached “strong agreement” after the first round. The remaining 28 recommendations, where no “strong agreement” was reached, were discussed in a face-to-face panel meeting during the ESPNIC conference in Salzburg, Austria (June 2019): 21 were rephrased for 2nd round of voting, one was designated as “no agreement,” and panel proposed deleting 6 recommendations as they were thought to be redundant after discussing all other recommendations (Fig. 1).

Finally, of the total 94 recommendations, 72 reached “strong agreement,” 20 “weak agreement,” and on 2 proposed recommendations “no agreement” was reached as summarized in “Supplementary file - Table 1.”

## Discussion and evidence for the recommendations

The details of technique, methods, reliability, search for published evidence, and references are provided in the online supplement (10.1186/s13054-020-03326-2). The commonly used parameters for hemodynamic monitoring in critically ill children are measurements of the blood pressure, central venous pressure (CVP), central venous oxygen saturation, cardiac output, serum lactate, pulmonary artery catheter, transpulmonary dilution, clinical signs, near-infrared spectroscopy, fluid responsiveness, microcirculation, and role of ultrasonography A brief summary of the evidence related to each sub-section has been summarized below.

### Clinical signs

Pediatric resuscitation courses (such as ETAT WHO, APLS) teach initial assessment of the shocked child well. Most caregivers will be familiar with the clinical signs and symptoms that help assess the hemodynamic status in children, including heart rate (HR), blood pressure, respiratory rate (RR), state of consciousness, diuresis, core and peripheral temperature, capillary refill time, and peripheral perfusion. Some of these parameters are age-dependent, and some can be altered by ambient temperature, pain, anxiety, and many other factors. The mission of the primary resuscitation team is to identify the shocked child in need for urgent intervention and treatment, usually with fluids and then inotropes or vasopressors in some combination.

All recommendations reached a high level of agreement, both in identifying children in need for treatment and in the limited value of clinical signs to guide hemodynamic treatment. There is a significant variability in clinicians’ abilities to assess hemodynamic clinical parameters at the bedside. Early signs of hemodynamic decompensation may be subtle and can be easily missed by the clinicians [14]. For these reasons, the frequent and trend evaluation of clinical signs are more important than a single specific determination. A combination of vital signs can be more useful to evaluate hemodynamic state than individual parameters [15].

Disappointingly, there is no good correlation between clinical assessment and invasive hemodynamic parameters, which only indicates that clinical parameters and invasive parameters do not measure the same compartment [16]. Hence, in hemodynamically unstable patients apart from frequent meticulous assessment, monitoring trends of several measurable clinical, biochemical, and monitoring parameters should be used to guide the therapy timely and accurately (Table 1).

**Table 1:** Recommendations on use of clinical examination and blood pressure measurement in hemodynamic monitoring in critically ill children

Sr No	Recommendation	Level of agreement
**Clinical signs**		
1.	There is no single clinical parameter that allows to evaluate the global hemodynamic status in children and, therefore, we recommend to analyze several parameters and make frequent assessments.	Strong agreement
2.	We recommend to perform a clinical assessment as the initial evaluation in all patients for the detection of hemodynamic alterations and to evaluate clinical signs periodically together with hemodynamic monitoring parameters in unstable patients.	Strong agreement
3.	We do not recommend to titrate hemodynamic therapy or fluid loading solely based upon clinical signs or a reduced urine output alone in unstable patients with the exception of the initial resuscitation phase.	Strong agreement
**Arterial blood pressure**		
4.	We recommend the use of intra-arterial blood pressure (IBP) over oscillometric blood pressure (OBP) measurement when a reliable blood pressure (BP) measurement is of importance or when fast changes in blood pressure need to be detected.	Strong agreement
5.	In children over 12 years of age we recommend a target blood pressure of ≥ 65 mmHg MAP (according to adults surviving sepsis guidelines) unless in children known to have prior hypertension.	Strong agreement
6.	We recommend not to use BP as the only therapeutic target in unstable children. The hemodynamic state should be evaluated integrating several clinical and hemodynamic parameters.	Strong agreement
7.	We recommend IBP monitoring in children in shock not responsive to initial fluid therapy or requiring vasopressor treatment, and hypertensive emergencies to control the effect of continuous invasive hypotensive drugs.	Strong agreement

### Arterial blood pressure

Blood pressure (BP) measurement is one of the most commonly used hemodynamic parameters for diagnostic and therapeutic decisions in critically ill children, not least due to ease of utilization and, if invasive, the additional benefit of arterial blood sampling, as well as continuous data sampling. Both a low and a high BP on admission are related to an increased mortality [17]. Accurate measurement of BP is considered essential for the diagnosis and treatment of hypertension as well as of hypotension, including various categories of hemodynamic shock [18, 19]. BP can be measured invasively but also by using several less reliable non-invasive methods [20].

The committee strongly agreed on the use of intra-arterial blood pressure (IBP) over oscillometric blood pressure (OBP) measurement when there is a need for reliable BP measurement in children with shock not responding to initial fluid therapy or requiring inotropes or vasoactive medication; in patients with intracranial hypertension and intracranial pressure monitoring to measure cerebral perfusion pressure; during major surgery and in children with malignant hypertension or other hypertensive emergencies and to monitor the effect of continuous intravenous vasoactive medications or inotropes. However, the clinical value of BP in guiding hemodynamic therapy was not appreciated equally among the panel members. Nevertheless, there was strong agreement that BP should not be used as the only therapeutic target in unstable children, so the hemodynamic state should be evaluated integrating BP with several clinical and additional hemodynamic parameters [21].

Optimal values for BP in healthy and critically ill children, including therapeutic thresholds, should be related to the clinical condition, age, sex, and body size [20–25]. There was only weak agreement concerning BP values in children under 12 years of age. In children over 12 years of age generally, we strongly recommend a target mean arterial pressure (MAP) of ≥ 65 mmHg, although in specific situations, the targeted BP may be higher such as when managing raised intracranial pressure.

### Central venous pressure (CVP)

The committee shared a strong common opinion regarding CVP. CVP should be measured as accurately as possible and be evaluated only as part of multi-modal hemodynamic monitoring to assess the intravascular volume and cardiac function [26, 27]. Isolated CVP measurement is of limited value but trends of CVP, both the value and the wave morphology, or change in CVP in response to fluid or vasoactive therapy may provide useful information about the overall hemodynamic status and cardiovascular physiology in critically ill children. Specifically, a rise or high levels of CVP should be avoided [28]. The committee agreed that CVP is not of great value in the initial treatment of critically ill children, but it can deliver important additional information in children with shock refractory to initial hemodynamic treatment; however, the use of CVP requires a good understanding of its limitations and pathophysiology of underlying disease process. For example, CVP should not be used as a sole parameter to guide fluid therapy [29–32].

### Central venous oxygen saturation measurement

Central venous oxygen saturation (ScvO_2_) approximates but does not equal mixed oxygen saturation (SmvO2). The normal ranges for ScvO2 and SmvO2 are 70–80% and 60–70%, respectively, in the setting of a normal aortic saturation [33, 34]. Trends between ScvO2 and SmvO2 are often interchangeable, although SmvO2 values are generally around 7–10% lower than ScvO2. A low ScvO_2_ typically indicates a mismatch between oxygen supply and utilization. Conversely, a normal, or high ScvO_2_ value, does not necessarily signify supply-demand adequacy, as tissue dysoxia (which may occur in sepsis) may cause an artificial elevation (or normalization) of ScvO_2_. Lastly, ScvO2 in isolation cannot be considered a surrogate of cardiac index/cardiac output [35]. However, there is some evidence that resuscitation in sepsis might be more beneficial when ScvO_2_ is incorporated in the treatment strategy [36].

The committee agreed that ScvO_2_ is an important parameter in unstable patients not responding to the first treatment and that its trend is helpful in hemodynamic management. However, we recommend that hemodynamic therapy should not be targeted solely based upon ScvO_2_ levels (Table 2).

**Table 2:** Recommendations on use of measurement of CVP, SCVO_2_, and prediction of fluid responsiveness in hemodynamic monitoring in critically ill children

Sr No	Recommendation	Level of agreement
**Central venous pressure**		
1.	We recommend to place the tip of a central venous catheter at the junction of the superior caval vein (SCV) and the right atrium to obtain an optimal central venous pressure (CVP) measurement or ScvO_2_ sample.	Strong agreement
2.	We recommend to measure CVP in all unstable patients refractory to initial hemodynamic treatment.	Strong agreement
3.	We recommend against the use of CVP to predict fluid responsiveness; Fluid loading should not be started solely based upon a low CVP.	Strong agreement
4.	An isolated CVP measurement is of limited value in clinical practice. However, trends in CVP may provide important information regarding changes in cardiovascular pathophysiology such as evolving right heart failure and an abrupt elevation in CVP upon fluid administration should raise suspicion of significant cardiac dysfunction.	Strong agreement
**Central venous oxygen saturation measurement**		
5.	We recommend to measure central venous oxygen saturation (ScvO_2_) in unstable patients not responding to the initial treatment. ScvO_2_ < 65% suggest a possible hemodynamic alteration; however, in sepsis, a normal or high ScvO_2_ may reflect mitochondrial dysfunction and mask hemodynamic alterations.	Strong agreement
6.	ScvO_2_ is not an adequate marker of cardiac index (CI).	Strong agreement
7.	We recommend against targeting hemodynamic therapy solely based upon ScvO_2._	Strong agreement
**Volume resuscitation and fluid responsiveness**		
8.	We recommend to observe the patient’s clinical situation, physical exam, and various perfusion indicators suggesting an inadequate CO (or oxygen transport) caused by hypovolemia before considering fluid loading.	Strong agreement
9.	In delivering a bolus of fluid, we recommend to administer a small bolus of fluid in a short time period while tracking changes in cardiac output, blood pressure and CVP, and when possible or available, to confirm fluid responsiveness before commencing fluid loading therapy.	Strong agreement
10.	We recommend alternative therapeutic strategies for hypotension management in fluid non-responders.**	Strong agreement
11.	We recommend to withhold fluid therapy in patients with an increasing CVP and no significant increase in blood pressure or cardiac output as a result of previous fluid therapy.	Strong agreement
12.	We recommend fluid therapy (with boluses 5–10 ml/kg) as part of early resuscitation in unstable patients guided by the effect on blood pressure and/or cardiac output.	Strong agreement

**Non-responders defined cases who had no rise in cardiac output (or stroke volume) as a result of volume resuscitation

### Volume resuscitation and fluid responsiveness

Volume resuscitation is one of the most commonly used therapeutic options. Nevertheless, excessive fluid administration may impair tissue perfusion even further by promoting edema and third-space fluid accumulation [6, 7, 37]. A rise in cardiac output (or stroke volume) as a result of volume resuscitation is called fluid responsiveness. To prevent unnecessary fluid administration, it could be beneficial to predict fluid responsiveness before the fluids are delivered. Unfortunately, there is no clear, simple, and proven method to predict fluid responsiveness in children. Static measures, mostly CVP, are not appropriate to test fluid responsiveness [29, 31]. The published evidence suggests that respiratory variation in aortic blood flow peak velocity {(ΔVPeak/velocity time integral (VTI)} is the most reliable indicator of fluid responsiveness, but only in ventilated children that fulfil various criteria [38]. Other dynamic methods, like passive leg raising test and liver pressure, have not been adequately assessed in children of all ages [39].

Due to the lack of simple bedside, available methods to determine fluid responsiveness and the risk of fluid overload with aggressive approach, the committee recommended the following: recurrent smaller fluid boluses (maximal 5–10 ml/kg) in a short-time interval in patients with hemodynamic instability while tracking changes in cardiac output, blood pressure, and CVP to confirm or assess fluid responsiveness. Furthermore, we strongly agreed to recommend withholding fluid therapy in patients with an increasing CVP and no significant increase in blood pressure or cardiac output as a result of previous fluid therapy. No specific recommendations regarding estimating fluid responsiveness can be made in patients with raised intracranial pressure or extracorporeal life support (ECLS).

### Echocardiography/ultrasonography

Cardiac ultrasound or functional echocardiography is non-invasive and easily available at the bedside in the intensive care setting, and it allows rapid evaluation of hemodynamic status in real time. It can be used to assess cardiac function and preload, to estimate cardiac output and fluid responsiveness, to measure pulmonary artery systolic pressure and serial assessment, and allows to evaluate response to therapy [10, 40]. Hence, it can be used, as an adjunct to other parameters, in children with hemodynamic instability to gain additional information required for making accurate clinical decisions such as myocardial failure, pulmonary hypertension, or cardiac tamponade [10]. It can help in understanding the pathophysiology of shock in children, and it can help in selecting timely, targeted specific, and right intervention [10, 41, 42].

The committee strongly recommended the use of cardiac ultrasound for hemodynamic evaluation of infants and children with hemodynamic instability. Since cardiac ultrasonography does not provide continuous measurements and is limited by a wide intra- and inter-observer variability [40, 43], we strongly recommend using serial assessments. However, we do not consider cardiac ultrasound as a tool for the routine hemodynamic monitoring in the intensive care setting (Table 3).

**Table 3:** Recommendations on the use of cardiac ultrasound and other methods to estimate cardiac output for hemodynamic monitoring in critically ill children

Sr No	Recommendation	Level of agreement
**Echocardiography/cardiac ultrasound**		
1.	We recommend to use cardiac ultrasound as an adjunct to gain additional information required for making accurate clinical decisions in infants and children with hemodynamic instability but not as a tool for routine hemodynamic monitoring in intensive care setting.	Strong agreement
2.	Cardiac ultrasound can help in diagnosing pulmonary hypertension and assessing severity of pulmonary hypertension, and in detecting cardiac tamponade.	Strong agreement
3.	We recommend monitoring of pulmonary artery pressure (PAP) using ultrasound with refractory shock states to exclude pulmonary hypertension. Cardiac ultrasound may help in identifying underlying pathophysiology of shock and choosing the right intervention based upon deranged hemodynamic physiology (preload, afterload, or cardiac function).	Strong agreement
4.	Cardiac ultrasound may help in assessing fluid responsiveness and we recommend using velocity time integral (VTI) across aortic valve for assessing fluid responsiveness rather than inferior vena cava collapsibility in mechanically ventilated infants and children.	Strong agreement
5.	We recommend using serial longitudinal assessments to assess response to therapy in patients with significant hemodynamic instability.	Strong agreement
**Cardiac output measurement and transpulmonary indicator dilution**		
6.	We recommend to use ultrasound/Doppler-based methods of estimating CO in stable patients, for the initial assessment of unstable patients and to decide if a more invasive method is required. When reliable absolute measurements of CO are deemed necessary, thermodilution (TPD) is the method of first choice.	Strong agreement
7.	In patients with a refractory shock when an accurate measurement of CO is needed, we recommend to use transpulmonary thermodilution (TPTD) or semi-invasive transpulmonary ultrasound dilution (TPUD).	Weak agreement
8.	We recommend to use invasive (and if possible continuous) CO monitoring in unstable post-operative patients after major (cardiothoracic) surgery, multiple trauma injuries or burns or patients with complex cardiopulmonary interactions.	Strong agreement
9.	We recommend against targeting fluid therapy based upon blood volumes measured with TPD or targeting hemodynamic therapy based upon lung water measurement to assess pulmonary edema in critically ill children.	Strong agreement
10.	Because of their intermittent measurement technique, TPD methods are not suitable for the detection of fast changes in CO unless used in conjunction with continuous trend monitoring using pulse contour analysis, calibrated by transpulmonary indicator dilution technology.	Strong agreement
**Pulmonary artery pressure**		
11.	We do not recommend to use pulmonary artery catheter (PAC) to measure CO in children. However, monitoring of left atrial pressure only in selected cardiac surgery patients or patients after lung transplant using a surgically inserted catheter can be helpful	Strong agreement

### Cardiac output monitoring and transpulmonary indicator dilution

Cardiac output (CO) is the product of heart rate and stroke volume. Stroke volume depends on preload, contractility, and afterload. The physical examination and simple commonly used hemodynamic parameters are the surrogate markers of cardiovascular well-being, but they do not provide a direct assessment of the cardiovascular hemodynamic status of the patient and clinical estimation of cardiac output has showed to be mostly inaccurate [14]. Hence, there seems to be a need of advanced hemodynamic monitoring to titrate therapies specifically when volume expansion or vasoactive drugs are delivered in order to improve cardiac output or systemic vascular resistance [44, 45]. In patients with refractory shock, when an effective and accurate measurement of CO is needed, the following methods may be used depending upon available resources and expertise: measurement of CO using transthoracic ultrasound (echocardiography) and transpulmonary dilution (TPD) [44–46]. Ultrasonography is non-invasive, easily available, and can provide a fairly accurate and serial estimation of cardiac output at the bedside to monitor the initial response to therapy [40, 47]. However, it requires specific skills and it is operator-dependent. Despite being the most reliable clinical method to measure cardiac output, the application of TPD in the clinical practice may be challenging because of resources, technical difficulties, or lack of expertise. It is invasive and not suited to emergency resuscitation. Only two methods are available in children below 40 kg: transpulmonary thermodilution (TPTD) by PiCCO (Pulsion Medical Systems, Germany) and transpulmonary ultrasound dilution (TPUD) by CO status (Transonic, USA) [44–46, 48]. Neither is in frequent use in ICU due to their intricate set-up, and particularly for PICCO, the risk to children’s vessels from relatively large femoral arterial catheter required. Moreover, because of their intermittent measurement technique, TPD methods are not suitable for the detection of rapid, frequent, and changes in hemodynamic status, as required in some critically ill children.

In the clinical practice, we recommend to use cardiac output ultrasound/Doppler methods for estimating CO in stable patients and the initial assessment of unstable patients. The committee did not reach to a strong agreement on the methods to estimate CO in children with refractory shock needing escalation of treatment and hemodynamic monitoring. There was a strong agreement that TPD methods are the most reliable but whether their use should be advised in situations needing escalation only reached a weak agreement. TPD methods also measure blood volumes and lung water, but the committee recommended against using these parameters for targeting hemodynamic goals. Measuring CO using PAC is not recommended.

Cardiac output can also be estimated at the bedside using other non-invasive methods like bioimpedance and bioreactance, pulse contour, and Doppler. Validation of these methods in critically ill children is sparse, and these methods are therefore not consensually viewed as accurate enough to estimate absolute values of CO in the intensive care setting in children [49]. However, they might provide a trend over time. In general, the committee strongly agreed that at the current time no recommendations regarding these methods can be given due to the limited experiences in critically ill children.

### Pulmonary artery pressure (PAP)

The pulmonary artery catheter (PAC) can provide continuous measurement of right atrial, PAP, measurement of CO, and pulmonary arterial occlusion pressure (wedge pressure). However, because of invasiveness and size, it is not used or recommended in intensive care clinical practice in children [50]. Similarly, left atrial pressure can be measured using surgically inserted left atrial catheters (LAC) [51, 52]. Still, alternative less invasive techniques are being used in children to estimate left atrial pressure in unstable patients and LAC or PAC are rarely used in today’s intensive care clinical practice [53, 54].

Because of the above, the committee recommends not to use a PAC in children to measure cardiac output or PAP in the ICU. Instead, transthoracic ultrasound echocardiography can be easily used to estimate PAP at the bedside non-invasively and it can provide serial assessment to monitor the response to therapy or disease process (as above). However, it should not be used to estimate PAP in patients with right ventricular (RV) failure [55]. For precise measurement of PAP, we recommend using the PAC only at the cardiac catheterization laboratory.

### Lactate measurement

Determination of blood lactate concentration is a cheap, fast, and easy bedside parameter that has demonstrated utility to predict the outcome or to trigger the need to intensify medical treatment [56]. The committee showed some variation in their approach to the use of lactate in children since 5 out of 10 recommendations needed a revision.

In critically ill patients or children with shock, early and serial lactate blood sampling from a reliable site such as a central venous or arterial indwelling catheter is recommended, though peripheral venous sampling with tourniquet time < 60 s is possible [57]. This is specifically recommended when the initial capillary lactate value is > 3.0 mmol/L [57–59]. Studies report an association between failure to normalize lactate levels to a certain threshold (3.0 ± 1.0 mmol/L) during the first 12 to 24 h of ICU admission, and adverse outcomes regardless of the reason for ICU admission [60, 61]. Experts could not agree on the use of lactate as part of a goal-directed approach and only weakly agreed on the approach to a persistent high lactate level. In the latter, lactate levels should always be used in conjunction with other clinical indicators of poor systemic perfusion and monitoring parameters. Persistently elevated lactate levels may reflect other mechanisms rather than those derived from poor tissue perfusion in shock and instead reflect aerobic glycolytic mechanisms including catecholamine administration or endogenous release [62] (Table 4).

**Table 4:** Recommendations on use of serum lactate, near infrared spectroscopy (NIRS), and microcirculation assessment for hemodynamic monitoring in critically ill children

Sr No	Recommendation	Level of agreement
**Serum lactate measurement**		
1.	We recommend to obtain a repeat blood sample from a reliable site when the lactate value of a capillary sample is higher than 3.0 mmol/L and to closely follow-up patients and intensify treatment until lactate values at least drop below 3.0 mmol/L, especially if other concerns regarding tissue hypoxia are present.	Strong agreement
2.	We recommend to interpret lactate levels always in conjunction with clinical indicators of poor systemic perfusion and monitoring parameters.	Strong agreement
**Near infrared spectroscopy**		
3.	Trend in NIRS values may provide valuable physiological information in children with hemodynamic instability but routine use in all children with hemodynamic instability is not recommended.	Strong agreement
4.	Near infrared spectroscopy (NIRS) can be useful during the peri-operative period after surgery for congenital heart defects; however, we recommend against the routine use of NIRS during non-cardiac surgery.	Weak agreement
**Microcirculation**		
5.	Many routinely used parameters like capillary refill, peripheral temperature, lactate, NIRS etc., reflect aspects of the hemodynamic condition, but they do not adequately reflect the microcirculation. Although central venous to arterial CO_2_ difference could provide additional insight into the microcirculatory condition, we recommend against its use to guide resuscitation in critically ill children	Strong agreement
6.	We recommend against routine microcirculation evaluation by video microscopy in stable children except those in clinical studies	Strong agreement

### Near-infrared spectroscopy

Near-infrared spectroscopy (NIRS) is a non-invasive, bedside technique to estimate regional capillary-venous hemoglobin saturation (rSO2). The mean baseline cerebral rSO_2_ is > 70% in healthy children. Infants and children with cyanotic heart disease may have a cerebral rSO_2_ between 46–57% [63–67]. Moreover, practitioners should be mindful about a considerable variability in NIRS values between commercially available devices. It has been observed that values measured in both monitors INVOS 5100-C® (Medtronic; Boulder, CO, USA) and Foresight Elite® monitor (CAS Medical Systems; Branford, CN, USA) are not interchangeable [68]. Although NIRS is mainly used to measure rSO2 in the brain, there are also reports of its use on other organs. In a study by Dabal et al. [69], it appears that renal NIRS and inferior vena cava desaturations precede rScO2 changes in the prediction of serious cardiovascular adverse events in patients after stage 1 Norwood palliation. Trend in NIRS values may provide valuable physiological information in children with hemodynamic instability although clear (cut-off) values and evidence of benefit are lacking.

The committee strived to define recommendations with regard to this subject and 6 out of 7 recommendations had to be redefined. As a result, the only strong recommendation was to advise against routine use of NIRS in all children with hemodynamic instability. Moreover, the committee agreed not to make recommendations regarding the use of NIRS while treating children in shock, post-cardiac arrest, post traumatic brain injury, and infants with hypoxic-ischemic encephalopathy. Lastly, there was no agreement on the clinical usefulness of a decline of cerebral rSO2 under 40–50% or a change in baseline of more than 20% [70].

### Microcirculation

Microcirculatory assessment by videomicroscopy using side-stream or incident dark field is expensive and not widely available. Currently, it does not allow for assessment of rapid circulatory changes during resuscitation [71]. No studies have defined the normal values of microcirculation in children outside the neonatal period but do report that vascular density seems to decrease with age [72]. So far, published studies have not defined target values of microcirculatory parameters in critically ill children [72–77]. At this point in time, the committee recommends its use only for research purposes.

The committee also states that many routinely used parameters like capillary refill, peripheral temperature, and lactate may reflect aspects of the hemodynamic condition but do not adequately reflect the microcirculation and cannot be used as such. Although central venous to arterial CO_2_ difference could provide additional insight into the microcirculatory condition, we currently recommend against its use to guide resuscitation in critically ill children.

### Limitations

We acknowledge the limitations of these recommendations as follows: (1) The most important limitation is the lack of high-quality evidence. These recommendations are based upon expert consensus and review of the published literature including experts’ opinions, which can involve subjective value judgments; (2) both lower and upper limits of age, from term infant > 37 weeks and postnatal age > 4 weeks to 18 years, are artificial—to avoid overlapping with neonatal and adult population-specific guidelines; and (3) some of these recommendations may not be appropriate for low-resource settings and may not be applicable in all settings requiring hemodynamic monitoring in children because of their limited availability or expertise.

Nevertheless, despite these limitations, the committee members believe that these are consensual expert recommendations based upon literature review and rigorous standardized process of developing expert consensus—followed the DELPHI approach, a well-established standardized approach (DELPHI approach)—to reach consensus in such circumstances of limited published evidence to develop evidence-based guidelines.

### Future directions

The committee recognizes that there is an important lack of knowledge and evidence concerning hemodynamic monitoring in children. There is a great need for (1) studying the relationship between measured parameters and end-organ perfusion, and (2) evaluating the clinical efficiency and patient outcome when therapy is guided by specific monitoring technologies.

## Conclusions

Cardiovascular instability is common in children admitted to pediatric intensive care. Multiple-organ dysfunction is commonly associated with cardiovascular derangements in patients with shock and carries high mortality. Effective hemodynamic monitoring can help in identifying cardiovascular instability early and choosing the appropriate targeted therapy timely. Currently, with the exception of management of shock, there are no published HD monitoring guidelines for critically ill children, and the published evidence remains scarce. These are therefore the first expert consensus recommendations for HD monitoring in critically ill children with hemodynamic instability. These recommendations can help clinicians in their clinical practice and may become the frame for future research aiming at providing strong data for evidence-based guidelines in this field.

## Supplementary information

**Additional file 1: Table 1** Summary of expert-consensus recommendations for hemodynamic monitoring in critically ill neonates and children. **Supplementary information.** Supplement documents providing background information for the 12 defined subjects.

## Data Availability

Supplementary material provided and attached with manuscript submission. Supporting data on consensus development and voting available if required.
