# Supplementary material for: Recommendations for hemodynamic monitoring for critically ill children—expert consensus statement issued by the cardiovascular dynamics section of the European Society of Paediatric and Neonatal Intensive Care (ESPNIC)
Source: Crit Care. 2020 Oct 22;24:620. doi: 10.1186/s13054-020-03326-2 (PMC7579971; doi:10.1186/s13054-020-03326-2)
Supplement: Supplementary file 1 — Additional file 1: Table 1 Summary of expert-consensus recommendations for hemodynamic monitoring in critically ill neonates and children. Supplementary information. Supplement documents providing background information for the 12 defined subjects. [file 13054_2020_3326_MOESM1_ESM.docx]

**Table 1: Summary of expert-consensus recommendations for hemodynamic monitoring in critically ill neonates and children**

| **Sr No** | **Recommendation** | **Level of agreement** |
| --- | --- | --- |
| **1. Clinical signs** | | |
| CS1 | We recommend to perform a clinical assessment as the initial evaluation in all patients for the detection of hemodynamic alterations and thereupon decide on the subsequent monitoring, diagnostic tests and initial treatment | Strong agreement |
| CS2 | There is no single clinical parameter that allows to evaluate the global hemodynamic status in children and, therefore, we recommend to analyze several parameters and make frequent assessments | Strong agreement |
| CS3 | We recommend to interpret heart rate and respiratory rate with respect to the age or height of patient, sex, temperature and other influencing factors | Strong agreement |
| CS4 | We recommend to measure thermal gradient and capillary refill time to evaluate peripheral perfusion | Strong agreement |
| CS5 | In unstable patients we recommend to evaluate clinical signs periodically together with hemodynamic monitoring parameters | Strong agreement |
| CS6 | We do not recommend to titrate hemodynamic therapy based upon clinical signs alone in unstable patients | Strong agreement |
| CS7 | We do not recommend fluid loading solely based upon clinical signs with exception of the initial resuscitation phase | Strong agreement |
| CS8 | We do not recommend fluid loading solely based upon a reduced urinary output | Strong agreement |
| **2. Arterial blood Pressure** | | |
| BP1 | We recommend the use of intra-arterial blood pressure (IBP) over oscillometric blood pressure (OBP) measurement when a reliable blood pressure (BP) measurement is of importance | Strong agreement |
| BP2 | We recommend the use of IBP over OBP when fast and accurate changes in blood pressure need to be detected | Strong agreement |
| BP3 | In children under 12 years of age we recommend a target blood pressure (preferably mean arterial pressure (MAP)) during shock and after return of spontaneous circulation from cardiac arrest higher than P5 for the age and sex, and if possible, around the P50, unless uncontrolled hemorrhage due to trauma is present. We advise to take blood flow parameters into account and avoid the overuse of vasoconstrictors when guiding hemodynamic therapy using BP | Weak agreement |
| BP4 | In children under 12 years of age we do not recommend a target MAP > 65 mmHg unless under very specific conditions (e.g. intracranial hypertension) | Strong agreement |
| BP5 | In children over 12 years of age we recommend a target blood pressure of >= 65 mmHg MAP (according to adults surviving sepsis guidelines) unless in children known to have prior hypertension | Strong agreement |
| BP6 | We recommend not to use BP as the only therapeutic target in unstable children. The hemodynamic state should be evaluated integrating several clinical and hemodynamic parameters | Strong agreement |
| BP7 | We recommend to measure IBP in children after major surgery that could produce hemodynamic, respiratory or neurologic alterations or risk of bleeding | Strong agreement |
| BP8 | We recommend IBP monitoring in children in shock not responsive to initial fluid therapy or requiring vasopressor treatment | Strong agreement |
| BP9 | We recommend to use IBP in malignant hypertension or other hypertensive emergencies to control the effect of continuous invasive hypotensive drugs. Oral or intermittent intravenous drugs can be monitored using OBP | Strong agreement |
| BP10 | We recommend to use IBP in patients with intracranial hypertension to measure cerebral perfusion pressure (by subtracting IBP from intra cranial pressure) and control the effects of the therapy | Strong agreement |
| BP11 | We recommend to use IBP in patients on extracorporeal membrane oxygenation (ECMO) | Strong agreement |
| **3. Serum lactate measurement** | | |
| LAC1 | We recommend prompt point of care measurement of lactate or analysis in the laboratory according to local laboratory instructions | Strong agreement |
| LAC2 | We recommend to obtain a repeat blood sample from a reliable site (central venous, arterial or peripheral venous with a time of tourniquet use shorter than 60 secs) when the lactate value of a capillary sample is higher than 3.0 mmol/L | Strong agreement |
| LAC3 | We recommend to closely follow up patients with increased lactate levels until lactate values at least drop below 3.0 mmol/L, especially if other signs of tissue hypoxia are present | Strong agreement |
| LAC4 | We recommend to interpret lactate levels always in conjunction with clinical indicators of poor systemic perfusion and monitoring parameters | Strong agreement |
| LAC5 | We recommend to closely follow up and eventually intensify medical treatment in unstable patients with concerns regarding tissue hypoxia and lactate levels (> 3.0 mmol/L) | Strong agreement |
| LAC6 | We recommend to use goal-directed medical therapy in patients admitted after open heart surgery based on serial blood lactate values obtained in short periods of time (4 hours in children, contemplate 1 hour in neonates), considering 5 mmol/L as a cut-off value | No agreement |
| LAC7 | In children with septic shock and persistently high levels of lactate we recommend to intensify medical treatment (when possible) | Weak agreement |
| LAC8 | In children treated with extracorporeal life support with persistently high levels of lactate, we recommend to intensify medical or mechanical treatment | Weak agreement |
| **4.** **Central venous pressure** | | |
| CVP1 | We recommend to place the tip of a central venous catheter at the junction of the superior caval vein (SCV) and the right atrium to obtain an optimal central venous pressure (CVP) measurement | Strong agreement |
| CVP2 | We recommend the use of a short catheter with a semi-rigid wall connected with a transducer and electronic monitor to record CVP continuously | Strong agreement |
| CVP3 | We recommend to measure CVP in all unstable patients refractory to initial hemodynamic treatment | Strong agreement |
| CVP4 | We recommend against the use of CVP to predict fluid responsiveness. Therefore, fluid loading should not be started solely based upon a low CVP | Strong agreement |
| CVP5 | An abrupt elevation in CVP upon fluid administration should raise suspicion of significant cardiac dysfunction | Strong agreement |
| CVP6 | An isolated CVP measurement is of limited value in clinical practice. However, trends in CVP may provide important information regarding changes in cardiovascular pathophysiology such as evolving right heart failure | Strong agreement |
| CVP7 | CVP is not a reliable parameter to assess right ventricular function | Weak agreement |
| **5. Central venous oxygen saturation measurement** | | |
| ScvO_2_ 1 | We recommend to measure central venous oxygen saturation (ScvO_2_) in unstable patients not responding to the initial treatment | Strong agreement |
| ScvO_2_ 2 | ScvO_2_ >65%* (and arterial to venous difference less than 30%) is acceptable in children, and a sustained drop in ScvO_2_ (or increase in arterio-venous difference) may reflect inability of the cardiovascular system to respond to an increased demand or decreased supply | Weak agreement |
| ScvO_2_ 3 | ScvO_2_ values may differ depending upon the site of catheter tip: we recommend placing the central catheter tip at the junction of SVC and right atrium | Strong agreement |
| ScvO_2_ 4 | ScvO_2_ is not an adequate marker of cardiac index (CI) | Strong agreement |
| ScvO_2_ 5 | When ScvO_2_ is < 65% there is a possible hemodynamic alteration. However, in sepsis a normal or high ScvO_2_ may reflect mitochondrial dysfunction and mask hemodynamic alterations | Strong agreement |
| ScvO_2_ 6 | We recommend against targeting hemodynamic therapy solely based upon ScvO_2_ | Strong agreement |
| **This only applies for normal arterial saturation* | | |
| **6. Echocardiography / Ultrasonography** | | |
| US1 | We recommend against using cardiac ultrasound for routine hemodynamic monitoring in intensive care setting but in infants and children with hemodynamic instability it should be used as an adjunct to gain additional information required for making accurate clinical decisions | Strong agreement |
| US2 | Cardiac ultrasound can be reliably used in neonates and children with cardiac tamponade | Strong agreement |
| US3 | Cardiac ultrasound can help in diagnosing pulmonary hypertension and assessing severity of pulmonary hypertension | Strong agreement |
| US4 | Cardiac ultrasound may help in identifying underlying pathophysiology of shock and choosing the right intervention based upon deranged hemodynamic physiology (preload, afterload or cardiac function) | Strong agreement |
| US5 | Cardiac ultrasound performed by adequately trained intensivists can help in assessing global cardiac function qualitatively on visual inspection and also semi-quantitatively | Weak agreement |
| US6 | Cardiac ultrasound may help in assessing fluid responsiveness: we recommend using velocity time integral (VTI) across aortic valve for assessing fluid responsiveness rather than inferior vena cava collapsibility in mechanically ventilated infants and children | Strong agreement |
| US7 | We recommend serial longitudinal assessment to assess response to therapy in patients with significant hemodynamic instability | Strong agreement |
| **7.** **Cardiac output measurement and transpulmonary indicator dilution** | | |
| CO1 | We recommend against the use of PAC for measuring CO in children as the first-choice method | Strong agreement |
| CO2 | In patients with a refractory shock when an accurate measurement of CO is needed, we recommend to use transpulmonary thermodilution (TPTD) or semi-invasive transpulmonary ultrasound dilution (TPUD) | Weak agreement |
| CO3 | We recommend to use ultrasound/doppler based methods of estimating CO if TPTD or TPUD are not available or do not match the conditions to be used | Strong agreement |
| CO4 | We recommend to use ultrasound/doppler based methods of estimating CO for the initial assessment of unstable patients, to decide if a more invasive method is needed or in stable patients | Strong agreement |
| CO5 | We cannot give any recommendations regarding other non-invasive methods due to the limited experiences in critically-ill children | Strong agreement |
| CO6 | We recommend that cardiac index (CI) should be maintained above 3.5 L/min/m^2^, otherwise titrated to ensure adequate end organ support | Strong agreement |
| CO7 | In young children the use of uncalibrated continuous arterial pressure-based CO monitoring is not recommended | Strong agreement |
| CO8 | We recommend to use invasive (and if possible continuous) CO monitoring in unstable post-operative patients after major (cardiothoracic) surgery, multiple trauma injuries or burns or patients with complex cardiopulmonary interactions | Strong agreement |
| CO9 | We recommend to use invasive and or calibrated continuous CO monitoring in patients with high intrathoracic pressure that “threatens” the hemodynamic status (like severe PARDS) for titrating hemodynamic and ventilatory therapy | Weak agreement |
| TPD1 | We recommend against the routine use of transpulmonary dilution (TPD) in children | Strong agreement |
| TPD2 | Blood volumes measured with TPD reflect volume status in children, however we recommend against targeting fluid therapy based upon these parameters | Strong agreement |
| TPD3 | Lung water measurement may provide a physiological insight into the amount of pulmonary edema in critically ill children. However, we recommend against targeting hemodynamic therapy based upon this parameter | Strong agreement |
| TPD4 | TPD methods can be useful for indicating cardiac or non-cardiac shunts | Weak agreement |
| TPD5 | TPD methods are the most accurate methods available at the bedside for measuring CO in children | Weak agreement |
| TPD6 | When reliable absolute measurements of CO are deemed necessary TPD is the method of first choice | Strong agreement |
| TPD7 | Because of their intermittent measurement technique, TPD methods are not suitable for the detection of fast changes in CO unless used in conjunction with continuous trend monitoring using pulse contour analysis, calibrated by transpulmonary indicator dilution technology | Strong agreement |
| TPD8 | In unstable children TPD measurements may be advantageous. However, the risk of femoral arterial access when using transpulmonary thermodilution (TPTD) method and complicated measurements must be weighed against the potential benefit | Strong agreement |
| **8.** **Pulmonary artery pressure** | | |
| PAC1 | Transthoracic echocardiography is reliable enough to estimate systolic pulmonary artery pressure (SPAP) at the bedside in most patients when tricuspid valve regurgitation is present and in absence of severe right ventricle (RV) failure | Weak agreement |
| PAC2 | We recommend using pulmonary artery catheters (PAC) for measurement of (gold standard) pulmonary arterial pressure (PAP) only during cardiac catheterization or, in selected cardiac surgery patients, using surgically inserted catheters | Strong agreement |
| PAC3 | We do not recommend to use a PAC for the measurement of cardiac output (CO) | Strong agreement |
| PAC4 | We do not recommend to use a PAC for the measurement of pulmonary artery wedge pressure | Strong agreement |
| PAC5 | We recommend the monitoring of left atrial pressure only in selected cardiac surgery patients or patients after lung transplant using a surgically inserted catheter | Strong agreement |
| PAC6 | We do not recommend non-invasive techniques using cardiac ultrasound to estimate left atrial pressure | Strong agreement |
| PAC7 | We recommend monitoring of PAP using ultrasound in young patients with refractory shock states to exclude pulmonary hypertension | Strong agreement |
| PAC8 | Transthoracic echocardiography can be a useful tool to estimate SPAP in patients on VV or VA ECMO | Weak agreement |
| **9. Volume resuscitation and fluid responsiveness** | | |
| FR1 | We recommend to observe the patient’s clinical situation, physical exam and various perfusion indicators suggesting an inadequate CO (or oxygen transport) caused by hypovolemia before considering fluid loading | Strong agreement |
| FR2 | We recommend, when possible or available, to confirm fluid responsiveness before commencing fluid loading when hypovolemia is suspected | Strong agreement |
| FR3 | In delivering a bolus of fluid we recommend to administer a small bolus of fluid in a short time period while tracking changes in cardiac output, blood pressure and CVP | Strong agreement |
| FR4 | We recommend alternative therapeutic strategies for hypotension management in fluid non-responders** | Strong agreement |
| FR5 | We recommend against delivering a fluid bolus based on static measures, particularly CVP | Strong agreement |
| FR6 | We recommend to withhold fluid therapy in patients with an increasing CVP and no significant increase in blood pressure or cardiac output as a result of previous fluid therapy | Strong agreement |
| FR7 | We recommend fluid therapy (with boluses 5-10 ml/kg) as part of early resuscitation in unstable patients guided by the effect on blood pressure and / or cardiac output | Strong agreement |
| FR8 | No specific recommendations regarding estimating fluid responsiveness can be made in patients with raised intracranial pressure or extracorporeal life support (ECLS) | Weak agreement |
| ***Non-responders defined cases who had no rise in cardiac output (or stroke volume) as a result of volume resuscitation.* | | |
| **8. Near infrared spectroscopy** | | |
| NIRS1 | The mean baseline cerebral capillary-venous hemoglobin saturation (rSO_2_) is >70% in healthy children and those with an acyanotic heart disease (similar to the adult population). Infants and children with cyanotic heart disease have a mean cerebral rSO_2_ between 46-57%* | Weak agreement |
| NIRS2 | Cerebral rSO_2_ less than 40% or a significant drop from the baseline may be associated with hypoxic-ischemic neural injury | No agreement |
| NIRS3 | It is recommended that the cerebral probe should be placed on the right and or left side of the forehead | Weak agreement |
| NIRS4 | Near infrared spectroscopy (NIRS) can be useful during the peri-operative period after surgery for congenital heart defects | Weak agreement |
| NIRS5 | We recommend against the routine use of NIRS during non-cardiac surgery | Weak agreement |
| NIRS6 | We cannot make recommendations regarding the use of NIRS while treating children in shock, post-cardiac arrest, post traumatic brain injury and infants with hypoxic-ischemic encephalopathy | Weak agreement |
| NIRS7 | Trend in NIRS values may provide valuable physiological information in children with hemodynamic instability but routine use in all children with hemodynamic instability is not recommended | Strong agreement |
| **Normal range described is while using INVOS NIRS monitor and practitioners should be mindful that different devices and sensors provide different values – we recommend checking normal values for the device being used in clinical practice* | | |
| **11. Microcirculation** | | |
| MICRO1 | In addition to monitoring blood pressure and cardiac output assessment of microcirculation should be considered in children with shock | Weak agreement |
| MICRO2 | Many routinely used parameters like capillary refill, peripheral temperature, lactate, NIRS etc. reflect aspects of the hemodynamic condition but do not adequately reflect the microcirculation and cannot be used as such | Strong agreement |
| MICRO3 | We recommend against routine microcirculation evaluation by video microscopy in stable children except those in clinical studies | Strong agreement |
| MICRO4 | We recommend to evaluate the microcirculation by video microscopy in various types of patients with hemodynamic compromise or complicated clinical course only for research purposes | Strong agreement |
| MICRO5 | We recommend the use of microcirculation measurement technologies such as sidestream dark field (SDF) or incident dark field (IDF) in sublingual area in critically ill children that are deeply sedated to evaluate microcirculation | Strong agreement |
| MICRO6 | We recommend to use the recommendations of 2017 adult consensus to the acquisition and analysis of video microscopy (SDF or IDF cameras) | Strong agreement |
| MICRO7 | Although central venous to arterial CO_2_ difference could provide additional insight into the microcirculatory condition, we recommend against its use to guide resuscitation in critically ill children | Strong agreement |

**Supplement documents providing background information for the 12 defined subjects**

Literature search

A literature search was performed until May 2019, using PubMed, Embase, Up-to-date databases etc. Search was performed using MESH terms and free words.

**1. Clinical signs**

**Short description of the parameter/method**

One of the fundamental objectives of the initial hemodynamic assessment is to provide an early identification of patients who have hemodynamic compromise, in order to establish appropriate monitoring and treatment. For this reason, it is very important to define the clinical signs and symptoms that may help to identify patients not equipped with advanced hemodynamic monitoring tools, with incipient hemodynamic alterations as early as possible. The initial basic clinical parameters that allow a hemodynamic assessment are heart rate (HR), respiratory rate (RR), state of consciousness, diuresis, core and peripheral temperature, and peripheral perfusion assessment.

**Short overview of the reliability of the method**

**Heart rate and cardiac auscultation**

Heart rate (HR) and stroke volume are the determinants of cardiac output in children. Children, especially newborns and small infants, more easily increase HR than stroke volume, to maintain cardiac output. However, in children the heart rate is very frequently affected by non-hemodynamic alterations and, therefore, it is a very sensitive but not very specific parameter [1]. Table 1 summarizes some fundamental factors that can modify the heart rate in childhood. Amongst these, temperature is an important factor that determines heart rate in children [2].

Sinus tachycardia is one of the earliest signs of hemodynamic alteration in children. In contrast, bradycardia may be a late sign and should always be a warning signal of severe hemodynamic alteration with a risk of cardiac arrest. However, not only the absolute value of the heart rate must be taken into account, but also its variability, hence in children at risk of hemodynamic alteration a frequent or continuous assessment of the heart rate is necessary. Cardiac auscultation, in addition to the specific findings if the hemodynamic alteration is secondary to a cardiac pathology, may show tachycardia, gallop rhythm or rhythm disturbances.

**Respiratory frequency and pulmonary auscultation**

The respiratory rate in children is inversely proportional to age. The respiratory rate is not only altered by respiratory diseases. Hemodynamic alterations can produce initially an increase in respiratory rate, and in case of metabolic acidosis, an increase of the depth of the breathing, as well as changes on the breathing pattern.

In severe hemodynamic alteration, bradypnea can occur, which, like bradycardia, is a sign of imminent risk of cardiac arrest. Pulmonary auscultation in children with hemodynamic abnormalities is often initially normal. Crackles be heard in cases of heart failure with secondary pulmonary edema as well as pneumopathies concomitant with septic shock. The respiratory frequency can be monitored continuously and simply, besides clinically, by impedance, with the ECG monitoring electrodes. It is necessary to place the electrodes sufficiently separated in both sides of the thorax to ensure adequate detection of respiratory movements.

**Pulse palpation**

Systolic blood pressure (SBP) can be estimated approximately, with palpation of the central pulses (carotid, brachial or femoral) and peripheral pulses (radial or pedal)[2]. The amplitude of the pulse reflects the difference between the SBP and diastolic blood pressure (DBP) and indirectly the stroke volume. In general, it is considered that if pulses are palpated radial or tibial, it is assumed that the SBP is greater than 90 mmHg. If the peripheral pulses are not palpated but the central ones are, the SBP will be between 50 and 90 mmHg, and when the central pulses are not palpated, the SBP will be less than 50 mmHg. However, pulse palpation remains subjective as it is a difficult maneuver even for experienced professionals, particularly in emergency situations. In addition, peripheral pulses can also be weakened by peripheral vasoconstriction secondary to fever, cold or anxiety, or else as a compensatory mechanism.

**Peripheral perfusion of the skin**

The cutaneous vascular bed plays an important role in thermoregulation. Under normal conditions, skin coloration and temperature of the trunk and limbs are similar. When the skin is well perfused, it remains dry, rosy and warm both centrally and peripherally (fingers and toes). By contrast, in situations of vasoconstriction, the toes and hands become cold and pale and the skin becomes speckled irregularly, (cutis marmorata). The skin marmorata is the result of a heterogeneous vasoconstriction of the small skin vessels and reflects a poor peripheral perfusion. Skin vasoconstriction can be observed in many occasions including a low ambient temperature, stress, emotional reactions, increased respiratory effort but also circulatory shock.

**Capillary refill time (CRT)**

The assessment of the capillary refill time is used to estimate the distal skin perfusion. It is done by applying a pressure during 5 seconds at ambient temperature that produces whitening in an area of the skin (fingers, feet or the presternal area) [3–5]. After releasing the pressure, the coloring should return in less than or equal to 2 or 3 seconds [6]. The increase in capillary refill time may reflect poor skin perfusion is an early sign of shock and is more elongated the more advanced the state of shock [4]. A novel digital videography with a video camera could better measure CRT than conventional CRT, but yet again, it is challenging to generalize its clinical use [7].

These skin signs are valuable, since they appear in the earliest phases of shock, indicating that the peripheral vasoconstrictive response has started. CRT is also useful to evaluate dehydration in children [8]. It is also useful in assessing patient evolution, since its progression in a centripetal fashion indicates worsening, persistence of skin signs fosters poor prognosis, and conversely, improvement of CRT suggests adequate therapeutic responsiveness [9, 10].

Peripheral perfusion monitoring is simple, non-invasive, can be performed anywhere, and correlates with lactate levels and venous saturation [11], multiorgan failure and prognosis [12]. In addition, in patients with septic shock, peripheral perfusion changes may be independent of systemic hemodynamic changes such as BP and cardiac output [13, 14]. It appears that changes in peripheral perfusion reflect changes in regional vasomotor tone rather than systemic blood flow [9]. However, the assessment of peripheral perfusion is subjective and there may be significant variations between observers [14], it is not specific and it correlates weakly to stroke volume index in children with septic shock [15]. Moreover, although this may not be reproducible in pediatrics, in adults with septic shock a resuscitation strategy targeting normalization of capillary refill time, compared with a strategy targeting serum lactate levels, did not reduce mortality [16]. Another sign of peripheral vasoconstriction is the presence of a line of separation between cold and hot skin at the level of any part of the limbs. This line progresses proximally if vasoconstriction worsens and distally if peripheral perfusion improves.

**Central and peripheral temperature. Thermal gradient**

Central temperature can be measured in the armpit, forehead, mouth, auditory canal. esophagus or rectum. The peripheral temperature is usually measured in the big toe. The peripheral temperature depends preferably on the blood flow to the peripheral circulation and therefore, decreases when peripheral vasoconstriction occurs (due to hemodynamic or hypothermic alteration). The differential temperature or thermal gradient is the difference between the central and peripheral temperature. Normal values are less than 4-5˚C depending on age and ambient temperature. The differential temperature is an objective measure of vasoconstriction during shock [9]. The core body temperature is maintained to preserve the perfusion of the vital organs at the expense of decreasing the distal temperature, resulting in an increase in the thermal gradient.

**Cerebral perfusion**

Signs of cerebral hypoperfusion depend on the intensity and onset time and always indicate severity. When hypoperfusion sets in abruptly there may be loss of muscle tone, generalized seizures and loss of consciousness. When the cerebral hypoperfusion is established slowly, neurological symptoms also appear more insidiously, as alterations of the level of consciousness (irritability, confusion, lethargy).

**Urinary output**

In the normal individual and in the absence of renal insult, diuresis correlates well with hemodynamic status. Oliguria and anuria in critically-ill patients are indicators of inadequate renal perfusion in patients in shock and urinary output is useful to monitor both its progression and to evaluate therapeutic effectiveness. On the other hand, the hemodynamic state is only one of many factors influencing diuresis and a low urinary output does not always indicate shock or hypovolemia. In children with severe burns, fluid therapy based upon advanced hemodynamic monitoring has been deemed more efficient than that based upon classical parameters including urinary output [17].

**Clinical signs of heart failure**

Patients with right-sided heart failure may have pleural effusions, hepatomegaly, ascites and peripheral edema. However, jugular engorgement is inconsistent in childhood.

In left-sided heart failure, clinical signs are more related to the above description of poor peripheral perfusion or shock, in the setting of tachycardia, progressive lactic acidosis, and impeding respiratory distress due to lung edema

Table 3 shows the clinical alterations that appear in situations of initial and established shock.

**Normal or target values**

Table 2 summarizes the normal values of heart and respiratory rate in childhood.

Specific tables should be used [18–21]. The wide range of normality of these parameters in each age must be taken into account. For this reason, the frequent evaluation of trends is more important than a specific determination.

**The estimated clinical usefulness**

Clinical hemodynamic assessment is the easiest and most important method for the initial evaluation of the hemodynamic state. The fundamental advantages of clinical hemodynamic assessment are that it is accessible, quick and easy and some parameters are affected early in the course of disease while others can be measured continuously allowing an adequate assessment of the evolution and effect of the established treatments. A combination of vital signs can be more useful to evaluate hemodynamic state than individual parameters [22].

The major drawbacks of most hemodynamic parameters of clinical assessment though, are that they vary with age, they are not specific and may be altered by many non-hemodynamic causes such as fever, pain, irritability. Moreover, there is no good correlation between clinical assessment and invasive hemodynamic assessment [23–27]. But this absence of hemodynamic coherence only indicates that clinical parameters and invasive parameters do not measure the same hemodynamic compartment rather than the fact that clinical parameters are not useful [28].

Last but not least, there is a significant variability in clinicians’ abilities to assess hemodynamic clinical parameters at the bedside, with the further limitation that early signs of hemodynamic decompensation may be subtle and easily omitted by clinicians [29].

**TABLE 1. FACTORS THAT AFFECT THE HEART RATE**

| **Factor** | **Bradycardia** | **Tachycardia** | **Arrythmia** |
| --- | --- | --- | --- |
| Sympathetic stimulation |  | X | X |
| Vagal stimulation | X |  | X |
| Catecholamines |  | X | X |
| Stress, pain |  | X |  |
| Fever |  | X |  |
| Hypothermia | X |  | X |
| Hypotension |  | X |  |
| Hypovolemia |  | X |  |
| Activity, crying |  | X |  |
| CNS injury | X | X | X |
| Hypothyroidism | X |  | X |
| Hyperthyroidism |  | X | X |
| Hyperkalemia |  |  | X |
| Hypokalemia |  |  | X |
| Hypercalcemia | X |  | X |
| Hypocalcemia |  | X | X |
| Hypermagnesemia |  |  | X |
| Hypomagnesemia |  |  | X |
| Digital | X |  | X |
| Phenothiazines |  |  | X |
| Phenytoin |  |  | X |
| Propranolol | X |  | X |
| Quinidine | X |  | X |
| Tricyclic antidepressants |  | X | X |
| Verapamil | X |  | X |
| Amiodarone | X |  |  |
| Flecainide |  |  | X |

**TABLE 2. NORMAL HEART RATE AND RESPIRATORY RATE ACCORDING TO THE AGE**

| **Age** | **HR (bpm)** | **RR (rpm)** |
| --- | --- | --- |
| Premature | 140 - 160 | 40 - 60 |
| Term newborn | 140 - 160 | 40 - 60 |
| 6 months | 120 - 160 | 30 - 60 |
| 1 year | 100 - 140 | 24 - 40 |
| 2 years | 90 - 140 | 24 - 40 |
| 4 years | 80 -110 | 22 - 34 |
| 6 years | 75 - 100 | 18 - 30 |
| 8 years | 75 -100 | 18 - 30 |
| 10 years | 75 -100 | 18 - 30 |
| 12 years | 75 -100 | 18 - 30 |
| > 14 years | 60 - 90 | 12 - 16 |

HR: heart rate. RR: respiratory rate.

**TABLE 3. CLINICAL SIGNS AND SHOCK**

|  | **Initial Compensated** | **Established** |
| --- | --- | --- |
| **HR**  **Pulse**  **BP** RR **Skin** Capillary refill **Diuresis**  **CNS** | Tachycardia  Normal  Normal or increased  Normal  Pale, cold, sweaty  Slow (> 2 seconds)  Oliguria  Irritability. Agitation | Tachycardia  Weak  Hypotension  Tachypnea  Mottled, cold, peripheral cyanosis (acrocyanosis)  Very slow  Oliguria or anuria  Stupor o coma |

# HR: heart rate, BP: blood pressure, RR: respiratory rate, CNS: central nervous system.

**References for clinical signs**

1. Lemson J, Nusmeier A, Hoeven JG van der (2011) Advanced Hemodynamic Monitoring in Critically Ill Children. Pediatrics 128:560–571.

2. Davies P, Maconochie I (2009) The relationship between body temperature, heart rate and respiratory rate in children. Emerg Med J 26:641.

3. Gorelick MH, Shaw KN, Baker MD (1993) Effect of ambient temperature on capillary refill in healthy children. Pediatrics 92:699–702

4. Fleming S, Gill P, Jones C, et al (2015) Validity and reliability of measurement of capillary refill time in children: a systematic review. Arch Dis Child 100:239.

5. Raichur DV, Aralihond AP, Kasturi AV, Patil DH (2001) Capillary refill time in term neonates: bedside assessment. The Indian Journal of Pediatrics 68:613–615

6. Carcillo JA (2012) Pediatr Crit Care Med

7. Shavit I, Brant R, Nijssen-Jordan C, et al (2006) A novel imaging technique to measure capillary-refill time: improving diagnostic accuracy for dehydration in young children with gastroenteritis. Pediatrics 118:2402–2408.

8. Caruggi S, Rossi M, Giacomo CD, et al (2018) Pediatric Dehydration Assessment at Triage: Prospective Study on Refilling Time. Pediatric Gastroenterology Hepatology Nutrition 21:278–288.

9. Genderen ME van, Bommel J van, Lima A (2012) Monitoring peripheral perfusion in critically ill patients at the bedside. Curr Opin Crit Care 18:273–279.

10. Leonard PA, Beattie TF (2004) Is measurement of capillary refill time useful as part of the initial assessment of children? Eur J Emerg Med 11:158–163.

11. Raimer PL, Han YY, Weber MS, et al (2011) A Normal Capillary Refill Time of < 2 Seconds is Associated with Superior Vena Cava Oxygen Saturations of > 70%. J Pediatrics 158:968–972.

12. Fleming S, Gill P, Jones C, et al (2015) The Diagnostic Value of Capillary Refill Time for Detecting Serious Illness in Children: A Systematic Review and Meta-Analysis. Plos One 10:e0138155.

13. Tibby SM, Hatherill M, Murdoch IA (1999) Capillary refill and core–peripheral temperature gap as indicators of haemodynamic status in pediatric intensive care patients. Arch Dis Child 80:163.

14. Lobos A-T, Lee S, Menon K (2011) Capillary refill time and cardiac output in children undergoing cardiac catheterization. Pediatr Crit Care Me 13:136–140.

15. Vos-Kerkhof E de, Krecinic T, Vergouwe Y, et al (2016) Comparison of peripheral and central capillary refill time in febrile children presenting to a pediatric emergency department and its utility in identifying children with serious bacterial infection. Arch Dis Child 102:17-21.

16. Hernández G, Ospina-Tascón GA, Damiani LP, et al (2019) Effect of a Resuscitation Strategy Targeting Peripheral Perfusion Status vs Serum Lactate Levels on 28-Day Mortality Among Patients With Septic Shock. Jama 321:654–11.

17. Genderen ME van, Paauwe J, Jonge J de, et al (2014) Clinical assessment of peripheral perfusion to predict postoperative complications after major abdominal surgery early: a prospective observational study in adults. Crit Care 18:R114.

18. Fleming S, Thompson M, Stevens R, et al (2011) Normal ranges of heart rate and respiratory rate in children from birth to 18 years of age: a systematic review of observational studies. Lancet 377:1011–1018.

19. O’Leary F, Hayen A, Lockie F, Peat J (2015) Defining normal ranges and centiles for heart and respiratory rates in infants and children: a cross-sectional study of patients attending an Australian tertiary hospital pediatric emergency department. Arch Dis Child 100:733.

20. Sepanski RJ, Godambe SA, Zaritsky AL (2018) Pediatric Vital Sign Distribution Derived From a Multi-Centered Emergency Department Database. Frontiers Pediatrics 6:66.

21. Bonafide CP, Brady PW, Keren R, et al (2013) Development of Heart and Respiratory Rate Percentile Curves for Hospitalized Children. Pediatrics 131:e1150–e1157.

22. Thompson M, Coad N, Harnden A, et al (2009) How well do vital signs identify children with serious infections in pediatric emergency care? Arch Dis Child 94:888–893.

23. Egan JR, Festa M, Cole AD, et al (2005) Clinical assessment of cardiac performance in infants and children following cardiac surgery. Intens Care Med 31:568–573.

24. Razavi A, Newth CJL, Khemani RG, et al (2016) Cardiac output and systemic vascular resistance: Clinical assessment compared with a noninvasive objective measurement in children with shock. J Crit Care 39:6–10.

25. Tibby SM, Hatherill M, Marsh MJ, et al (1997) Clinical validation of cardiac output measurements using femoral artery thermodilution with direct Fick in ventilated children and infants. Intensive Care Medicine 23:987–991

26. Hiemstra B, Koster G, Wiersema R, et al (2019) The diagnostic accuracy of clinical examination for estimating cardiac index in critically ill patients: the Simple Intensive Care Studies-I. Intens Care Med 45:1–11.

27. Hiemstra B, Eck RJ, Keus F, Horst ICC van der (2017) Clinical examination for diagnosing circulatory shock. Curr Opin Crit Care 23:293–301.

28. Erdem Ö, Kuiper JW, Tibboel D (2016) 28. Erdem 2016 Best Practice & Research Clinical Anaesthesiology.pdf. Best Pract Res Clin Anaesthesiol 30:499–510.

29. Tibby SM, Hatherill M, Marsh MJ, Murdoch IA (1997) Clinicians’ abilities to estimate cardiac index in ventilated children and infants. Arch Dis Child 77:516–518.

**2. Arterial blood pressure**

**Short description of the parameter/method**

Arterial Blood Pressure (ABP) is dependent on cardiac output and systemic vascular resistance (SVR). The relation is reflected by the law of Ohm (ABP = CO x SVR) [1]. The regulation of BP is a complex process affected by many factors including the baroreflex, the renin-angiotensine-aldosterone-system (RAAS), sympathetic tone, blood viscosity and many others. ABP is one of the most often used parameters for diagnostic and therapeutic decisions in the pediatric intensive care unit (PICU). ABP measurements in children should be interpreted with regard to the clinical condition, age, sex and body size in order to judge whether the BP is normal or not [1][2].

**Short overview of the reliability of the method**

Blood pressure can be measured invasively but also by using several non-invasive methods.

**Non-invasive blood pressure (NIBP) using oscillometric methods**

Although the auscultatory blood pressure measurement still remains the gold standard for measuring ABP, the oscillometric technique is more commonly used in PICU and during anesthesia because of the frequent need for repeated BP monitoring [2]. Oscillometric ABP monitoring offers the advantage of intermittent, automatic and direct measurement of systolic (SAP), diastolic (DAP) and mean arterial pressure (MAP) [2]. Oscillometric devices calculate ABP from oscillations based on maximum buckling of the artery underneath the cuff, which is nearly equal to the mean arterial pressure. Subsequently, systolic arterial pressure (SAP) and diastolic arterial pressure (DAP) are calculated from this mean by device-specific algorithms or estimated from the start or stop of oscillations in the cuff [2][3].

Oscillometric devices are designed to measure the ABP with a cuff around the upper arm; both cuffs that are too narrow and cuffs that are too short will lead to falsely high ABP measurements. A cuff bladder which is too large will lead to an underestimation of ABP. According to the most widely known recommendations on cuff selection and the nomogram for ABP in children, the ABP cuff bladder should be wide enough to cover three quarters of the upper arm length (from the acromion to the olecranon) or should equal 40% of the mid-upper arm circumference [4].

Regrettably, oscillometric methods are not standardized, algorithms differ among manufactures and devices, and also few oscillometric monitor devices have been validated for (small) children [2]. Moreover, over inflation of automated cuffs can cause discomfort and subsequently child movement, resulting in inaccurate measurements [5]. Outside the normotensive range, the oscillometric ABP is higher during hypotension and lower during hypertension compared with the invasive ABP and doppler ultrasound methods [5–7]. In particular when vasoactive drugs are used oscillometric ABP could underestimate the SAP [8]. Furthermore, before treating hypotension, it might be useful to repeat oscillometric ABP measurements since a single measurement may give false results [6].

For clinical use of oscillometric ABP it is advised 1) to measure noninvasive blood pressure using the oscillometric ABP method at the upper arm (when possible). 2) to use an arm cuff of width equal to 40 % of the upper arm circumference. 3) to measure oscillometric ABP in multiple extremities if ABP is non-concordant with the clinical situation of the patient. 4) to repeat the oscillometric ABP measurement at least 2 times before starting fluid or vasoactive treatment for hypotension. 5) to change the cuff of oscillometric ABP device if ABP is non-concordant with the clinical situation of the patient.

In conclusion, oscillometric ABP can be used to adequately monitor ABP in normotensive patients. However, oscillometric ABP might underestimate the ABP during hypertension and overestimate ABP during hypotension. Because of the intermittent character, oscillometric ABP is also less reliable for detecting fast changes in ABP. For this reason, oscillometric ABP is less suitable to replace invasive ABP as method to guide diagnosis and the management in critically-ill children with an unstablehemodynamic situation [5, 5, 8, 9].

**Other non-invasive devices**

Some non-invasive devices are developed to measure ABP continuously in the wrist or fingers [10–12]. Although promising, there are reliability issues specifically in hypotensive situations. These devises are not commercially available for small children.

**Invasive arterial blood pressure**

The invasive ABP is the method closest to the gold standard Doppler ultrasound blood pressure measurement [7]. Direct measurement of arterial blood pressure requires cannulation of an artery with a fluid filled catheter and a pressure measurement transducer connected to a monitoring device. Holt et al. evaluated the quality of the invasive measurement by using morphologic criteria: the waves were considered good when they showed the systolic upstrokes, systolic peak pressure, systolic decline, dicrotic notch, diastolic run off, and end-diastolic pressure [7]. The transducer should be at the level of the 4th intercostal space at the mid-axillary line, calibrated to ambient pressure and ensuring absence of kinking or air bubbles in the tubing and the system [13]. In general, it is necessary to connect the transducer to a 300 mmHg pressure bag to run at 3 ml/hour to maintain continuous flow through the artery in order to prevent clotting. However lower flow, driven by infusion pumps is also acceptable.

The most used locations for placement of intra-arterial catheters for measuring IBP in children are the radial and the femoral artery but other sites are also possible (brachial or posterior tibial artery). However, depending on multiple factors BP in peripheral arteries may be different from BP in the femoral artery [14].

The arterial wave displayed on the monitoring device can significantly be influenced by the measuring system. System reliability is best when the catheters and tubing are stiff, the volume of the fluid is small, the number of stopcocks limited, and the length of the connecting tubing is limited. Furthermore, the system must be optimally damped. If the invasive ABP system is over or under damped the measured ABP is underestimated or overestimated respectively [8, 13, 13, 15].

An artifactual increase in systolic pressure (overshoot) and the decrease in diastolic pressure are indicative of a “resonance artifact”. Several modalities used for zeroing and transducer leveling during invasive ABM measurement may result in clinically significant overestimation of measured mean ABP pressure values [16]. There are specific transducers that increase the damping coefficient without affecting other properties of the system and they may limit errors due to artifacts [17].

The signal may be overdamped typically due to clots, air bubbles, or kinks in the arterial catheters [17]. When these artifacts occur, morphologic criteria may be inappropriate and evaluation of the damping condition of the system can be done using a flush test. A small volume of fluid is rapidly infused into the system, and the subsequent waveforms are recorded and used to calculate the natural frequency (how rapidly the system oscillates after a stimulus) and amplitude ratio (or damping coefficient); how quickly the system comes to rest due to frictional forces after a stimulus [17]. Moreover, invasive ABP can be difficult to obtain in some young patients and it could be associated with important complications as hemorrhage, vessel injury and infection [6]. Complications are related to size of catheter and duration of use [14].

For clinical use of invasive ABP: 1) the radial artery access is the first invasive location for the insertion of the intra-arterial catheter in children. 2) the femoral artery access serves as second choice for the insertion of the intra-arterial catheter in children. 3) The femoral artery can be used as primary when specific catheters are needed (e.g. PiCCO catheter). In all cases perfusion of the leg should be monitored closely. 4) for highest reliability the damping condition of the arterial blood pressure system should be evaluated by observing the pressure wave on the monitor screen and performing a fast-flush test when possible.

The most important indications for using invasive ABP are continuous monitoring of blood pressure and/or performing frequent blood tests.

**Normal or target values**

Normal values of ABP in healthy and critically-ill children are dependent on age, sex body proportion [18–20]. The fifth percentile cutoff for systolic ABP estimation of PALS guidelines (70 +2 x age in years) is often used and relatively accurate for critically-ill children from 1 to 10 years of age[19]. However, some authors suggested that these current values for hypotension may need to be adjusted for patient height and clinical condition [20]. For older children with septic shock probably a MAP > 65 mmHg seems adequate reflecting the adult surviving sepsis guidelines [21]. Algorithms concerning threshold values for hypotension do not always overlap [22]. Furthermore, the relationship between blood pressure and organ perfusion and subsequently the relationship between hypotension and organ failure is unclear. Therefore, it is unclear what the clinical relevance of certain low levels of blood pressure are [23][24]. However, changes in blood pressure during the course of illness or as a result of treatment are probably of greater importance. Underscoring the need for a blood pressure measurement system that delivers reliable continuous or semi-continuous blood pressure values.

**The estimated clinical usefulness**

Both a low and a high systolic ABP on admission are related to an increased mortality [25][26]. Blood pressure is one of the most used parameters to evaluate the physiologic hemodynamic state in critically-ill children. Accurate measurement of ABP is essential for the diagnosis and treatment of hypertension as well as of hypotension, including various categories of hemodynamic shock [5, 6][27]. Blood pressure, mainly mean arterial pressure, is the most used indirect parameter related with organ perfusion.

However, hypotension can be a late sign of shock because a decrease in cardiac output can be compensated by peripheral vasoconstriction to maintain ABP. Many factors, not only depending of the disease, could influence ABP in critically-ill children [28]. For example, in children on mechanical ventilation SAP variation significantly increases with elevated tidal volume [29].

**References for arterial blood pressure**

1. Lemson J, Nusmeier A, Hoeven JG van der (2011) Advanced Hemodynamic Monitoring in Critically Ill Children. Pediatrics 128:560–571.

2. Stergiou GS, Boubouchairopoulou N, Kollias A (2017) Accuracy of Automated Blood Pressure Measurement in Children. Hypertension 69:1000–1006.

3. Michard F, Sessler DI, Saugel B (2018) Non-invasive arterial pressure monitoring revisited. Intens Care Med 44:2213–2215.

4. Children NHBPEPWG on HBP in (2004) The Fourth Report on the Diagnosis, Evaluation, and Treatment of High Blood Pressure in Children and Adolescents. Pediatrics 114:555–576

5. Gupta P, Goel D (2018) Blood Pressure Measurement in Critically-ill Children: Where do we Stand? Indian pediatrics 55:289–291

6. Ray S, Rogers L, Noren DP, et al (2017) Risk of over-diagnosis of hypotension in children: a comparative analysis of over 50,000 blood pressure measurements. Intens Care Med 43:1540–1541.

7. Holt TR, Withington DE, Mitchell E (2011) Which pressure to believe? A comparison of direct arterial with indirect blood pressure measurement techniques in the pediatric intensive care unit. Pediatr Crit Care Me 12:e391–e394.

8. Joffe R, Duff J, Guerra GG, et al (2016) The accuracy of blood pressure measured by arterial line and non-invasive cuff in critically ill children. Crit Care 20:177.

9. Marouane A, Cornelissen EAM, Nusmeier A, Bootsma‐Robroeks CMHHT (2019) Oscillometric and intra‐arterial blood pressure in children post‐kidney transplantation: Is invasive blood pressure measurement always needed? Pediatr Transplant 23:e13309.

10. Wankum PC, Thurman TL, Holt SJ, et al (2004) Validation of a noninvasive blood pressure monitoring device in normotensive and hypertensive pediatric intensive care patients. Journal of Clinical Monitoring and Computing 18:253–263

11. Lemson J, Hofhuizen CM, Schraa O, et al (2009) The reliability of continuous noninvasive finger blood pressure measurement in critically ill children. Anesthesia Analgesia 108:814–821.

12. Hofhuizen CM, Lemson J, Hemelaar AEA, et al (2010) Continuous non-invasive finger arterial pressure monitoring reflects intra-arterial pressure changes in children undergoing cardiac surgery. Bja Br J Anaesth 105:493–500.

13. Krishna BV, Das S, Sen S (2018) Correlation Between Blood Pressure Measurement by Non-invasive and Invasive Methods in Critically-ill Children. Indian pediatrics 55:297–300

14. Cho HJ, Lee SH, Jeong IS, et al (2017) Differences in perioperative femoral and radial arterial blood pressure in neonates and infants undergoing cardiac surgery requiring cardiopulmonary bypass. J Pediat 94:76–81.

15. Romagnoli S, Ricci Z, Quattrone D, et al (2014) Accuracy of invasive arterial pressure monitoring in cardiovascular patients: an observational study. Crit Care 18:644.

16. Jacq G, Gritti K, Carré C, et al (2015) Modalities of Invasive Arterial Pressure Monitoring in Critically Ill Patients. Medicine 94:e1557-6.

17. Romagnoli S, Romano SM, Bevilacqua S, et al (2011) Dynamic response of liquid-filled catheter systems for measurement of blood pressure: precision of measurements and reliability of the Pressure Recording Analytical Method with different disposable systems. J Crit Care 26:415–422.

18. Eytan D, Goodwin AJ, Greer R, et al (2018) Distributions and Behavior of Vital Signs in Critically Ill Children by Admission Diagnosis&ast; Pediatr Crit Care Me 19:115–124.

19. Eytan D, Goodwin AJ, Greer R, et al (2017) Heart Rate and Blood Pressure Centile Curves and Distributions by Age of Hospitalized Critically Ill Children. Frontiers Pediatrics 5:52.

20. Banker A, Bell C, Gupta-Malhotra M, Samuels J (2016) Blood pressure percentile charts to identify high or low blood pressure in children. Bmc Pediatr 16:1–7.

21. Rhodes A, Evans LE, Alhazzani W, et al (2017) Surviving Sepsis Campaign. Crit Care Med 45:486–552.

22. Abdelrazeq S, Ray S, Rogers L, et al (2017) Age-associated blood pressure distributions in pediatric intensive care units differ from healthy children. Intens Care Med 44:1–3.

23. Kato R, Pinsky MR (2015) Personalizing blood pressure management in septic shock. Ann Intensive Care 5:41.

24. Haque IU, Zaritsky AL (2007) Analysis of the evidence for the lower limit of systolic and mean arterial pressure in children. Pediatr Crit Care Me 8:138–144.

25. Matettore A, Ray S, Harrison DA, et al (2019) Paediatric intensive care admission blood pressure and risk of death in 30,334 children. Intens Care Med 45:1482–1483.

26. Topjian AA, Telford R, Holubkov R, et al (2017) Association of Early Postresuscitation Hypotension With Survival to Discharge After Targeted Temperature Management for Pediatric Out-of-Hospital Cardiac Arrest. Jama Pediatr 172:1–3.

27. Cambiaso-Daniel J, Rontoyanni VG, Foncerrada G, et al (2018) Correlation between invasive and noninvasive blood pressure measurements in severely burned children. Burns 44:1–5.

28. Marlais M, Lyttle MD, Inwald D (2016) Ten concerns about blood pressure measurement and targets in pediatric sepsis. Intens Care Med 43:433–435. -7

29. Kühlwein E, Balmer C, Cannizzaro V, Frey B (2011) Determinants of arterial and central venous blood pressure variation in ventilated critically ill children. Intens Care Med 37:118–123.

**3. Serum lactate concentration**

**Short description of the parameter/method**

Blood lactate concentration elevates as a result of the metabolism of glucose during tissue hypoxia to produce energy (ATP), when cellular demands overcome oxygen availability and cellular oxygen consumption is dependent on oxygen delivery at a microcirculatory level.

Hyperlactatemia only results in an acidemia when the produced hydrogen ions cannot be recycled in the mitochondria, for example under anaerobic conditions. The elevation of lactate is not exclusively related to tissue hypoxia, but also to other aerobic mechanisms leading to an increased production or fluctuations in its clearance: increased aerobic glucose metabolism (stress, epinephrine administration), alkalosis (both metabolic and respiratory, via phosphofructokinase enzyme), lymphoma, mitochondrial dysfunction (in critically-ill children or other conditions), cytokine-mediated glucose uptake, lactate-containing buffer solutions (used in hemodialysis or hemofiltration), an inborn error of metabolism, fetal growth restriction and the use of some drugs (nucleoside reverse transcriptase inhibitors, biguanides, cyanides, methanol). It is not affected by the infusion of lactate-containing balanced crystalloids or by continuous renal replacement therapies. Lactate clearance is altered because of liver dysfunction (only in presence of high production), cardiac surgery postoperative period and sepsis. Paradoxical normal values can be observed if extensive tissue necrosis is present (as lactate production ceases) or when the increased lactate production cannot be mobilized towards the central circulation [1].

**Short overview of the reliability of the method if applicable**

Lactate measurement by point-of-care blood gas analyzers shows acceptable limits of agreement compared to central laboratory measurements, except when ethylene glycol intoxication is present (false elevation in blood gas analyzers). Leukocytosis or high hematocrit can produce in vitro elevation of lactate. A 15 minutes limit of storage, storage in ice, or fluoride containing tubes can limit this effect. Site of sampling (arterial, venous or capillary) seems to have acceptable agreement for low lactate values. Lack of agreement between capillary and arterial samples has been observed in the adult population except for normal values, whereas acceptable concordance has been reported in newborns [1]. Notably, there is a lack of studies involving children. In patients other than neonates, capillary samples should be used only as a screening tool. If lactate is higher than 2 mmol/L in a peripheral venous sample, advise to obtain an arterial sample has been reported [3]. Central venous samples have shown good agreement with arterial samples in pediatric unstable patients. Crystalloid solutions must be cleaned from central lines before drawing blood samples, especially if containing lactate as a buffer. There is almost no information regarding the tourniquet time needed to obtain a peripheral venous sample and the reliability of the lactate measured in it, with one study reporting acceptable values with a tourniquet time < 60 secs [4].

**Normal or target values**

Different units have been reported: 1 mmol/L equals 9 mg/dL of lactate. Although some laboratories have reported reference values of blood lactate between 0.5 and 1.5 mmol/L or 0.8 and 1.6 mmol/L, there is no clear and universally accepted definition cut-off level for hyperlactatemia. The majority of studies coincide to report the association between an absence of normalization of lactate levels to a certain threshold (3.0±1.0 mmol/L) during the first 12 to 24 hours of PICU admission with adverse outcomes (mortality, initiation of ECLS, impaired neurological development, multiple organ failure, and other), regardless of the reason for PICU admission: septic shock, cardiogenic shock, open heart surgery postoperative course, or others. This failure of improvement has been reported in many ways, such as thresholds at 12 or 24 hours after admission, lactate clearance (10% to 16% after 6 to 24 hours) or the time spent until normalization of lactate levels below 3.0±1.0 mmol/L. This time has been called “lactime” in some studies and has been reported as a powerful parameter to predict adverse outcomes that might advocate to increase medical therapy or to trigger the initiation of ECLS if maximal medical treatment fails [5-8].

**Short overview concerning the estimated clinical usefulness**

Determination of blood lactate concentration is a cheap, fast and easy bedside parameter that has demonstrated utility to predict the outcome or to trigger the need to intensify medical treatment [9]. However, it could be difficult to interpret as many non-anaerobic glycolysis mechanisms may produce hyperlactatemia. Simultaneous analysis of other parameters related to tissue hypoxia (central venous oxygen saturation, oxygen debt estimation, arterial to venous pCO_2_ difference, and peripheral perfusion) would help to avoid an overzealous and harmful resuscitation.

The extent of absolute hyperlactatemia at PICU admission is predictive of mortality, independent of illness severity or arterial pH and bicarbonate concentration and is comparable to the score of PRISM III [5]. A cut-off of 5.5 mmol/L at admission in PICU predicted mortality in a mixed population in a large prospective study including 1,109 children [5]. A lactate clearance below 16.4% of the initial value after 6h of PICU admission was related to mortality [7].

**Open heart surgery (OHS)**

Goal-directed medical therapy based on serial blood lactate values (measured every 4 hours or each hour in neonates) is associated with a marked reduction in mortality after pediatric OHS. Improvement is greatest in the highest risk patients: neonates (3.4% vs 12%), RACHS-1 categories 3-6 (3% vs 9%). The suggested cut-off level selected to increase medical therapy was 5.0 mmol/L, and 10 mmol/L to consider ECLS [8].

**Newborns**

Capillary blood lactate measurements in neonates showed a good agreement with arterial blood samples, however the difference between arterially and capillary sampled lactate concentration is increased in sepsis and during the use of vasopressors in newborns [2, 10]. Umbilical cord lactate after delivery was related to hypoxic-ischemic encephalopathy (HIE) in a systematic review including 12 studies (n=38,284), using a threshold of 3.2 mmol/L [10].

**Patients with severe sepsis**

A lactate level higher than 4.0 mmol/L or a failure to achieve a clearance of 10% after 24h in children presenting with systemic inflammatory response syndrome (SIRS) in the Emergency Department (ED) was useful to detect mortality in children with septic shock at PICU admission [11].

**Patients on Extracorporeal Life Support (ECLS) for primary respiratory disease**

In a prospective study including a combination of neonatal (n= 56) and pediatric (n=39) patients with primary respiratory disease (excluding primary cardiac disease) requiring ECLS, serial measurements of lactate were consistently higher in non-survivors with almost no overlap, specially between initiation of the therapy and up to day 4. A cut-off value of 2.5 mmol/L discriminated mortality exclusively in pediatric patients. Neonatal lactate values were not predictive of death in this study [12].

**References for serum lactate concentration**

1. Allen M (2011) Lactate and acid base as a hemodynamic monitor and markers of cellular perfusion. Pediatr Crit Care Med. 12:S43-9.
2. Fauchère JC, Bauschatz AS, Arlettaz R, Zimmermann-Bär U, Bucher HU (2002) Agreement between capillary and arterial lactate in the newborn. Acta Paediatr. 91:78-81.
3. Samaraweera SA, Gibbons B, Gour A, Sedgwick P (2017) Arterial versus venous lactate: a measure of sepsis in children. Eur J Pediatr. 176:1055-1060.
4. Gallagher EJ, Rodriguez K, Touger M (1997) Agreement between peripheral venous and arterial lactate levels. Ann Emerg Med. 29:479-483.
5. Bai Z, Zhu X, Li M, Hua J, Li Y, Pan J, Wang J, Li Y (2014) Effectiveness of predicting in-hospital mortality in critically ill children by assessing blood lactate levels at admission. BMC Pediatr. 28;14:83.
6. Hatherill M, McIntyre AG, Wattie M, Murdoch IA (2000) Early hyperlactataemia in critically ill children. Intensive Care Med. 26:314-8.
7. Kumar R, Kumar N (2016) Validation of lactate clearance at 6 h for mortality prediction in critically ill children. Indian J Crit Care Med. 20:570-574.
8. Rossi AF, Khan DM, Hannan R, Bolivar J, Zaidenweber M, Burke R (2005) Goal-directed medical therapy and point-of-care testing improve outcomes after congenital heart surgery. Intensive Care Med. 31:98-104.
9. Jansen TC, van Bommel J, Schoonderbeek FJ, Sleeswijk Visser SJ, van der Klooster JM, Lima AP, et al. LACTATE study group (2010) Early lactate-guided therapy in intensive care unit patients: a multicenter, open-label, randomized controlled trial. Am J Respir Crit Care Med. 182:752-761
10. Allanson ER, Waqar T, White C, Tunçalp Ö, Dickinson JE (2017) Umbilical lactate as a measure of acidosis and predictor of neonatal risk: a systematic review. BJOG. 124:584-594.
11. Choudhary R, Sitaraman S, Choudhary A (2017) Lactate clearance as the predictor of outcome in pediatric septic shock. J Emerg Trauma Shock. 10:55-59.
12. Buijs EA, Houmes RJ, Rizopoulos D, Wildschut ED, Reiss IK, Ince C, Tibboel D (2014) Arterial lactate for predicting mortality in children requiring extracorporeal membrane oxygenation. Minerva Anestesiol. 80:1282-93.

**4. Central venous pressure**

**Short description of the parameter/method**

Central venous pressure (CVP) is defined as the pressure of the blood in the thoracic vena cava near the right atrium. It is essentially equivalent to the pressure in the right atrium assuming no vena-cava obstruction (1,2).

It can be estimated by a central venous catheter placed in the intrathoracic portion of the superior vena cava (SVC), preferably at the junction of SVC and right atrium. Continuous CVP monitoring via electronic transducer is preferred (3).

**Short overview of the reliability of the method**

CVP can be evaluated as part of hemodynamic monitoring to assess intravascular volume and cardiac function. It is sometimes being used in clinical practice as a surrogate marker for cardiac preload and volume status in critically-ill children thereby guiding fluid resuscitation therapy, however, it may be misleading and unreliable to assess preload and fluid responsiveness (4,5).

**Normal or target values**

The range of normal value quoted in the literature is fairly wide with typical values, referred to the mid-axillary line as 2-10 cm H_2_0 (3). CVP depends upon the interaction between cardiac function and factors determining venous return to the heart. Body fluid volume, venous capacitance, venous compliance, heart rate, heart rhythm and conduction, structural defects of the heart, pericardial effusion, tamponade but also higher levels of PEEP are some of the major factors affecting CVP measurement (4).

**The estimated clinical usefulness**

Previously CVP was believed to be an indicator of right ventricular end-diastolic volume index and hence an indicator of preload. However, recent studies have shown that the use of CVP to assess preload and fluid responsiveness is far from infallible, and in fact it’s seriously flawed. Clinical studies have clearly demonstrated that ventricular volumes (right ventricular end-diastolic volume index, left ventricular end-diastolic area, global end-diastolic volumes) are unable to predict fluid responsiveness (4-7).  This could be explained from two important standpoints: 1) CVP is determined by the interaction of venous return and cardiac function. Therefore, physiological or pathophysiological significance of CVP can’t be interpreted without a simultaneous estimation of cardiac function and output, and 2) The relationship between CVP, right atrial pressure, right ventricular pressure, stroke volume and cardiac output is complex, dynamic and is dependent on multiple factors affecting preload, afterload, cardiac function and contractility, and myocardial compliance. Therefore, clinical usefulness of CVP in estimating right ventricular pressure has been questioned (2,8). It is also used for assisting in diagnosing and managing right heart failure.

Despite all the shortcomings, CVP measurement remains an important hemodynamic monitoring parameter in critically-ill children (2,4). However, its use in clinical practice needs good understanding of limitations and pathophysiology of disease process. Isolated CVP measurement is of limited value. The trends of CVP, both the value and the wave morphology, or change in CVP in response to fluid or vasoactive therapy may provide vital information about overall hemodynamic status of critically-ill children including right heart function and therapeutic responsiveness.

**References for central venous pressure**

1. Mark JB. Central venous pressure monitoring: clinical insights beyond the numbers. Journal of Cardiothoracic & Vascular Anesthesia 1991;5(2):163-73.

2. Magder, S. and F. Bafaqeeh. The clinical role of central venous pressure measurements. J Intensive Care Med 2007;22:44-51.

3. Friedman E, Grable E, Fine J. Central venous pressure and direct serial measurements as guides in blood-volume replacement. Lancet 1966;ii:609.

4. Magder S. Central venous pressure: A useful but not so simple measurement. Crit Care Med 2006;34(8):2224-7.

5. Marik PE, Cavallazzi R. Does the central venous pressure predict fluid responsiveness? An updated meta-analysis and a plea for some common sense. Crit Care Med 2013; 41(7):1774-81.

6. Gan H, Cannesson M, Chandler JR, Ansermino JM. Predicting fluid responsiveness in children: a systematic review. Anesth Analg 2013; 117(6):1380-92.

7. Roger C, Muller L, Riou B4 et al. Comparison of different techniques of central venous pressure measurement in mechanically ventilated critically ill patients. British Journal of Anaesthesia 2017; 118 (2): 223–31.

8. Kumar A, Anel R, Bunnell E, et al. Pulmonary artery occlusion pressure and central venous pressure fail to predict ventricular filling volume, cardiac performance, or the response to volume infusion in normal subjects. Crit Care Med 2004; 32(3): 691-9.

**5. Central venous oxygen saturation**

**Short description of the parameter/method**

The main goal of management in treating critically-ill patients in the PICU is to provide adequate perfusion and oxygenation to the tissues, which is achieved by maintaining a good balance between the delivery and consumption of oxygen. The venous oxygen saturation represents the oxygen reserve after oxygen extraction in the tissues and therefore reflects the competence of global tissue oxygenation. Imbalances in oxygen delivery and consumption can rapidly be identified and treated earlier with improved outcomes. This can be achieved by placing a central venous catheter in the superior vena cava (SCV) – at the junction of SCV and right atrium - and using spectroscopy. Ideally, mixed venous oxygen saturation (SmvO_2_) taken from the main pulmonary artery would be measured, however, this is not feasible in most of the patients in the neonatal and pediatric intensive care units (1-4). There is no continual difference between SmvO_2_ and central venous saturation (ScvO_2_). In clinical practice this oxygen balance can be monitored in real-time by measurement of ScvO_2_(5).

**Short overview of the reliability of the method**

The mixed venous oxygen saturation is determined by both the oxygen content and the magnitude of venous return via the superior vena cava (SVC), inferior vena cava (IVC) and coronary sinus (CS). The difference between SmvO2 and ScvO2 is influenced by the sampling site of ScvO2, the presence of left-to-right shunts, incomplete mixing of venous blood, level of cerebral oxygen extraction (anaesthesia, level of consciousness), redistribution of perfusion of the upper and lower body and myocardial oxygen consumption. ScvO_2_ trend correlated well with mixed oxygen saturation (SmvO_2_), however, it can’t be used as a surrogate of cardiac index / cardiac output (6).

Continuous measurement of ScvO2 has several benefits over intermittent measurements of ScvO_2_ by sampling central venous blood: results are available in real time providing early warning indicators, less risk of infection, decrease blood loss and hence avoids blood transfusion, convenient to patient and staff, and cost effective. Moreover, ScvO_2_ can help in guiding targeted therapy in patients with hemodynamic instability and sepsis (7,8).

**Normal or target values**

The normal values of ScvO_2_ and SmvO_2_ are 70-80% and 60-70%, respectively, in the setting of normal aortic saturation (1,9). Trends between ScvO_2_ and SmvO_2_ are almost interchangeable, although SmvO_2_ values are generally around 7-10% lower than ScvO_2_, and this gap widens in patients with shock state with redistribution of shock (1,10). Table 1

Table 1: Summary of central venous oxygen saturation value and impact of physiology

| **ScvO_2_ value** | **Physiology** |
| --- | --- |
| >70% | Normal oxygen extraction |
| 50-70% | Compensatory physiology because of decreased supply or increased demand |
| 30-50% | Limits of compensatory physiology (extraction) leading to onset of metabolic acidosis |
| 25-30% | Severe metabolic acidosis and hyperlactataemia |
| <25% | Cellular death |

**The estimated clinical usefulness**

Early indicator of change in ScvO_2_: When Scvo2 is measured continuously there may be variation of up to 5% because of change in physiology such as suction, handling, examination and other circumstances. Fluctuations (drop) of more than 5% from baseline usually reflect significant change and if the rate of recovery is slow then this may reflect inability of the cardiovascular system response to increased oxygen demand.

Low ScvO_2_ levels (ScvO_2_<70%) may be from decreased oxygen delivery (DO_2_) due to low cardiac output, decreased oxygen saturation and low hemoglobin concentration, or increased oxygen consumption or demand in sepsis, stress, pain, fever, shivering, seizures, or secondary to increased metabolic demand. Understanding the physiology and finding the underlying cause of low ScvO_2_ may help in instituting the specific targeted therapy timely and accurately (table 2). Therefore, continuous measurement of ScvO_2_ is crucial in hemodynamic monitoring of cardiovascular well-being and managing critically-ill children in PICU. It may help in improving patient outcomes (10,11).

Table 2: Causes of increased and decreased oxygen delivery (DO_2_) and oxygen consumption (VO_2_)

| Decreased SvO_2_ | Increased SvO2 |
| --- | --- |
| **Decreased Oxygen Delivery (DO_2_)**   - Decreased cardiac output - Anemia, hemorrhage - Hypoxia - Right-to-left shunting | **Increased Oxygen Delivery (DO_2_)**   - Increased cardiac output - Blood transfusion |
| **Increased Oxygen Consumption (VO_2_)**   - Fever, shivering - Pain, stress, agitation - Seizures - Increased metabolic demand (sepsis) - Respiratory failure | **Decreased Oxygen Consumption (VO_2_)**   - Sedation, analgesia, anesthesia - Muscle relaxation - Hypothermia - Mechanical ventilation |
|  | **Decreased Oxygen Extraction**   - Microcirculatory shunting (sepsis) - Cell death |

**References for central venous oxygen saturation**

1. Rivers EP, Ander DS, Powell D. Central venous oxygen saturation monitoring in the critically ill patient. Curr Opin Crit Care 2001; 7: 204–11.
2. Jean-Jaques R, Poète P, Bodin L, et al. Three mixed venous saturation catheters in patients with circulatory shock and respiratory failure. Chest 1990; 98(4): 954-8.
3. Peterson KJ. Measuring central venous pressure with a triple-lumen catheter. Critical Care Nurse 2012; 32(3): 62-4. doi: 10.4037/ccn2012554.
4. Whyte RK. Mixed venous oxygen saturation in the newborn. Can we and should we measure it? Scand J Clin Lab Invest 1990; 50(suppl 203):203–11.
5. Reinhart K, Kuhn HJ, Hartog C, Bredle DL. Continuous central venous and pulmonary artery oxygen saturation monitoring in the critically ill. Intensive Care Med 2004; 30(8): 1572-8.
6. Tibby SM, Murdoch IM. Monitoring cardiac function in intensive care. Arch Dis Child 2003; 88: 46–52.
7. Walley KR. Use of central venous oxygen saturation to guide therapy. Am J Respir Crit Care Med 2011; 184(5): 514-20. doi: 10.1164/rccm.201010-1584CI.
8. Nebout S, Pirracchio R. Should We Monitor ScVO(2) in Critically Ill Patients?. Cardiol Res Pract 2012; 2012: 370697. doi: 10.1155/2012/370697.
9. Bloos F, Reinhart K. Venous oximetry. Intensive Care Med 2005; 31(7): 911–913.
10. Crowley R, Sanchez E, Ho JK, Lee KJ, Schwarzenberger J, Marijic J, Sopher M, Mahajan A, Prolonged central venous desaturation measured by continuous oximetry is associated with adverse outcomes in pediatric cardiac surgery. Anesthesiology 2011; 115(5): 1033-43. doi: 10.1097/ALN.0b013e318233056e.
11. Shepherd SJ, Pearse RM. Role of central and mixed venous oxygen saturation measurement in perioperative care. Anesthesiology 2009; 111(3): 649-56. doi: 10.1097/ALN.0b013e3181af59aa.

**6. Cardiac ultrasound in children**

**Short description of the parameter/method**

Cardiac ultrasound, commonly referred to as echocardiography, is often used in the neonatal and pediatric intensive care setting for hemodynamic evaluation and it can also be used for intermittent hemodynamic monitoring. Transthoracic route is often used in the intensive care setting at the bedside and it is considered as the gold standard to diagnose causes of hemodynamic instability such as: pericardial effusion, cardiac tamponade, pulmonary hypertension and congenital or acquired heart defects (1-3).

Bedside ultrasound is non-invasive, portable and easily available in the intensive care setting, and its use in the neonatal and pediatric care arena is increasing specially to guide therapy. Studies have reported that clinical management was changed in 30-60% cases after the use of cardiac ultrasound at the bedside (1,3). However, it can be employed only intermittently and needs expertise to perform hemodynamic evaluation accurately, which makes it a less attractive hemodynamic monitoring tool. Last decade has seen an increased interest in training neonatal and pediatric intensivists in point of care ultrasound (POCUS) and international evidence-based guidelines on use of POCUS for critically-ill neonates and children issued by the POCUS Working Group of the European Society of Pediatric and Neonatal Intensive Care (ESPNIC) have been recently published (4).

**Short overview of the reliability of the method**

Transthoracic cardiac ultrasound is a non-invasive technique, which allows hemodynamic evaluation at the bedside in real time: the patient’s fluid responsiveness, the right and left ventricle cardiac output, the left and right cardiac function, to indirect estimate the pulmonary artery systolic pressure and the amount of shunt flow. This technique unfortunately does not allow to provide continuously measurements and is limited by a wide intra and inter-observer variability which can range between 12-22% (1,5). However, trends in hemodynamic changes can be easily evaluated using serial assessments, which is more useful than one off reading. Sometimes acquiring high quality images for precise functional assessment in ventilated children is challenging and this expertise may not be available 24/7 in all the intensive care settings. Moreover, its use is complementary to the clinical evaluation, biochemical markers, hemodynamic monitor and other tools being used for hemodynamic evaluation (1,6).

To assess fluid responsiveness in children with cardiac ultrasound, apart from the qualitative “eyeball method” for an under-filled heart (e.g. kissing wall left ventricle), there are other *quantitative* methods, including the inferior vena cava collapsibility index, the inferior vena cava distensibility index and the variation of the left ventricular outflow tract velocity time integral (VTI) (7-9). All of these have been evaluated in self-breathing adults and their role in children to assess fluid responsiveness under mechanical ventilation lacked predictive values.

The only reliable parameter which correlates with fluid responsiveness was the respiratory variation in the aortic outflow tract VTI (1). A variation of > 15% has been reported in children to have a high predictive value with sensitivity and specificity exceeding 90%. In absence of cardiac shunts, cardiac ultrasound allows the measurement of the stroke volume [cross sectional area (CSA) of the aortic vessel multiplied by the velocity time integral (VTI) of the blood flow across the CSA] and consequently the cardiac output. This assessment is prone to error as CSA is calculated by squaring the vessel’s diameter and like any other Doppler assessment VTI is dependent upon the angle of insonation. Nonetheless, in absence of cardiac shunts, its reliability has been validated in neonates and children when compared to gold standard assessment tools (such as MRI phase contrast and Fick hemodilution methods (1,6).

The assessment of the left ventricular (LV) function using the shortening fraction (SF) or the ejection fraction (EF) is part of the hemodynamic management in children with shock (10). This measurement was associated with an improvement in the patient outcomes of both neonates and children (10). However, in addition to the challenges in well-defined image acquisition, both SF and EF measurements are dependent on preload, afterload and ventricular septal deviation. These limitations have been overcome by the introduction of the tissue Doppler imaging (TDI) at the bedside which is relatively independent from the loading conditions and the ventricular geometry. However, all these Doppler measurements are dependent on the angle of insonation and good image acquisition which can be difficult in sick children needing mechanical ventilation and with limited ultrasound windows (11).

Assessment of pulmonary artery pressure (PAP) and right ventricular function is extremely important in the management of children with acute respiratory failure and acute pulmonary hypertension in the intensive care setting. Bedside point of care ultrasound (POCUS) can be reliably used to assess PAP at the bedside and it can be used to evaluate response to therapy (1). The geometry of the right ventricle makes it difficult to assess right ventricular function accurately. In addition to eyeballing, other techniques have been validated such as the tricuspid annular plane systolic excursion (TAPSE) and the Tissue Doppler Imaging to evaluate right ventricular systolic function (12).

**Normal or target values**

The clinical utility of LF function evaluation by measuring EF and SF has been established in multiple studies. The normal values for the shortening fraction range between 26-46%, while for the ejection the normal value is > 55%. We can consider as: mild LV dysfunction a patient with an EF between 40-55%; moderate LV dysfunction a patient with an EF between 30-39%; severe LV dysfunction a patient with an EF below 29% (1, 2,10).

**Short overview concerning the estimated clinical usefulness**

Bedside point of care cardiac ultrasound or functional echocardiography can provide accurate physiological and anatomical information to recognize hemodynamic instability in children. In fact, it should be considered an extension of the clinical examination and it should be used as an adjunct to the other existing tools being used for hemodynamic evaluation. It allows to assess cardiac function and fluid responsiveness, to estimate PASP and to evaluate the response to clinical interventions (responsiveness to inotropics, guidance in case of tamponade drainage, to mention some examples) (1, 4)

**References for cardiac ultrasound**

1. Singh Y. Hemodynamic evaluation on echocardiography in neonates and children. TINEC research paper. TINEC research paper. Frontiers in Pediatrics 2017; 5: 201. Doi:10.3389/fped.2017.00201

2. Tissot C, Singh Y, Sekarski N. Echocardiographic evaluation of ventricular function – for the neonatologist and pediatric intensivist. TINEC research paper. Frontiers in Pediatrics 2018; 6: 79.

3. Fraga M, Nishisaki A, Singh Y, et al. Moving beyond the stethoscope: diagnostic point-of-care ultrasound in pediatric practice. Pediatrics. 2019; 144(4): e20191402.

4. Singh Y, Tissot C, Fraga M, Yousef N, Cortes RM, Lopez J, et al. International evidence-based guidelines on Point of Care Ultrasound (POCUS) for critically ill neonates and children issued by the POCUS Working Group of the European Society of Paediatric and Neonatal Intensive Care (ESPNIC). Critical Care 2020; 24: 65.

5. de Boode W, van der Lee R, Horsberg B, Nestaas E, Dempsey E, Singh Y, et al. The role of Neonatologist Performed Echocardiography (NPE) in the assessment and management of neonatal shock. Pediatric Research 2018; 84: S57–S67. doi.org/10.1038/s41390-018-0081-1.

6. Singh Y, Katheria A, Vora F. Advances in diagnosis and management of hemodynamic instability in neonatal shock. Frontiers in Pediatrics 2018; 6:2. Doi: 10.3389/fped.2018.00002.

7. Desgranges F-P, Desebbe O, Pereira de Souza Neto E, Raphael D , Chassard d. Respiratory Variation in Aortic Blood Flow Peak Velocity to Predict Fluid Responsiveness in Mechanically Ventilated Children: A Systematic Review and Meta-Analysis. Paediatr Anaesth 2016; 26(1): 37-47. doi: 10.1111/pan.12803.

8. Pereira de Souza Neto E, Grousson S, Duflo F, Ducreux C, Joly H, Convert J, et al. Predicting fluid responsiveness in mechanically ventilated children under general anaesthesia using dynamic parameters and transthoracic echocardiography, BJA: British Journal of Anaesthesia 2011; 106: 856–864.

9. Choi DY, Kwak HJ, Park HY, et al. Respiratory Variation in Aortic Blood Flow Velocity as a Predictor of Fluid Responsiveness in Children After Repair of Ventricular Septal Defect. Pediatr Cardiol 2010; 31, 1166–1170.

10. Gasper HA, Morphy SS. The Role of Focused Echocardiography in Pediatric Intensive Care: A Critical Appraisal. BioMed Research International 2015, Article ID 596451.

11. Tissue Doppler velocity imaging and event timings in neonates: a guide to image acquisition, measurement, interpretation, and reference values. Pediatric Research 2018; 84: S18–S29. doi.org/10.1038/s41390-018-0079-8.

12. Jain A, Mohamed A, El-Khuffash A, et al. A comprehensive echocardiographic protocol for assessing neonatal right ventricular dimensions and function in the transitional period: normative data and z scores. J Am Soc Echocardiogr 2014; 27(12): 1293-304. doi:10.1016/j.echo.2014.08.018.

**7.** **Cardiac output**

**Short description of the parameter/method**

Cardiac output (CO) is the product of the heart rate and the stroke volume. Stroke volume depends on preload, contractility, and afterload. It represents a measure of heart function and is needed to calculate the oxygen delivery (CO x Hb x SaO2 = DO_2_) and oxygen consumption (VO_2_) to evaluate the general metabolism of the critically-ill patient. Often physical examination and simple hemodynamic parameters (heart rate, blood pressure and central venous pressure) do not reflect the real cardiovascular situation of the patient thus the need to use more advanced hemodynamic monitoring to titrate therapies [1-3]. Many methods of measuring CO have been developed and validated in children. These methods can be summarized into: indicator different-based methods (pulmonary artery thermodilution, transpulmonary thermodilution, lithium dilution, trans-pulmonary ultrasound dilution), ultrasound-based methods (transthoracic echocardiography, transesophageal echocardiography, doppler based methods (transesophageal Doppler, jugular non-invasive doppler), pulse contour methods, bioimpedance (thoracic electrical bioimpedance or whole-body electrical bioimpedance) and electrical velocimetry [3–9].

**Short overview of the reliability of the method if applicable**

Thermodilution using the pulmonary artery catheter has been used very commonly in adults to measure CO. However, it requires right heart catheterization which is challenging and complex in children due to small size and possible associated aberrant cardiopulmonary anatomy [1]. For these reasons, transpulmonary thermodilution technique has been the most used thermodilution method in children. Reliability in children can be guaranteed when these conditions are met: a) constant blood flow; b) zero or minimal loss of indicator; c) complete mixing of the indicator with blood; d) zero recirculation. Associated limitations are the size of the catheter, presence of intra-cardiac shunts and presence of any form of indicator-recirculation.

Transpulmonary ultrasound Dilution (TPUD) is a reliable method, even in the presence of shunts, but complex to use and time consuming, and requires specific training [8,10].

Ultrasound-based methods [4–6] use the (estimated) area of the aortic valve or the cross-sectional area of the descending aorta multiplied by the velocity time integral (for calculating stroke volume) and the heart rate to measure the CO [11]. Ultrasound-based methods have been studied in children against the gold standard methods (pulmonary artery catheter and the Fick technique) with conflicting results. These methods are operator-dependent and are not functional for continuous hemodynamic monitoring but may indicate a trend in CO [12, 13].

Pulse contour analysis of arterial pressure wave form and bio-impedance methods have shown controversial results regarding reliability.

Electrical velocimetry or electrical cardiometry represents a modified algorithm of precedent electrical impedance that transcutaneously detects changes in impedance with changes in erythrocyte orientation and flow peak velocity in the ascending aorta for continuous quantification of cardiac output [6, 7, 9]. This method has shown acceptable correlation when compared with ultrasound-based methods in children, but no data are available demonstrating good reliability compared to invasive methods.

**Normal or target values**

CO is a measure of total flow and it is influenced by patient size; it is therefore mandatory to quantify it as cardiac index (CI) (CI=CO/Body surface area). This removes the influence of different body sizes and provides a reference number suitable for children of different sizes. Normal values of CI have been established between approximately 3.5-5.5 L/min/m^2^ [1, 2].

**Short overview concerning the estimated clinical usefulness**

The evidence for the benefit of CO monitoring in children is limited, but what is known is the increased mortality associated to low CO syndromes [2].

Several clinical situations would benefit from CO monitoring in critically-ill children admitted to intensive care units such as: shock states (hypovolemic, septic, cardiogenic, distributive), congenital and acquired heart failure, multiple organ dysfunction syndrome, postoperative patients after major thoracic, neuro and abdominal surgery; multiple trauma injuries and burns; patients with complex cardiopulmonary interactions (pediatric ARDS patients requiring high PEEP level) and patients undergoing VV and VA ECMO. The most useful applications are 1) to establish whether a low blood pressure is caused by a low CO or a low systemic vascular resistance (or both); 2) whether the CO itself is high, low or in-between; 3) what the effect of interventions like fluid loading or vasoactive drugs is [14].

**References for cardiac output**

1. Nusmeier A, Hoeven JG van der, Lemson J (2014) Cardiac output monitoring in pediatric patients. Expert Rev Med Devic 7:503–517.

2. Proulx F, Lemson J, Choker G, Tibby SM (2011) Hemodynamic monitoring by transpulmonary thermodilution and pulse contour analysis in critically ill children. Pediatr Crit Care Me 12:459–466.

3. Fathi EM, Narchi H, Chedid F (2018) Noninvasive hemodynamic monitoring of septic shock in children. World J Methodol 8:1–8.

4. Saxena R, Durward A, Puppala NK, et al (2013) Pressure recording analytical method for measuring cardiac output in critically ill children: a validation study. Bja Br J Anaesth 110:425–431.

5. Alonso‐Iñigo JM, Escribá FJ, Carrasco JI, et al (2016) Measuring cardiac output in children undergoing cardiac catheterization: comparison between the Fick method and PRAM (pressure recording analytical method). Pediatr Anesth 26:1097–1105.

6. Singh Y (2017) Echocardiographic Evaluation of Hemodynamics in Neonates and Children. Frontiers Pediatrics 5:201.

7. Wurzer P, Branski LK, Jeschke MG, et al (2016) Transpulmonary Thermodilution Versus Transthoracic Echocardiography for Cardiac Output Measurements in Severely Burned Children. Shock 46:249–253.

8. Boehne M, Baustert M, Paetzel V, et al (2014) Determination of cardiac output by ultrasound dilution technique in infants and children: a validation study against direct Fick principle. Bja Br J Anaesth 112:469–476.

9. Chaiyakulsil C, Chantra M, Katanyuwong P, et al (2018) Comparison of three non-invasive hemodynamic monitoring methods in critically ill children. Plos One 13:e0199203.

10. Mohan UR, Britto J, Habibi P, et al (2002) Noninvasive Measurement of Cardiac Output in Critically Ill Children. Pediatr Cardiol 23:58–61.

11. Schubert S, Schmitz T, Weiss M, et al (2008) Continuous, non-invasive techniques to determine cardiac output in children after cardiac surgery: evaluation of transesophageal Doppler and electric velocimetry. J Clin Monitor Comp 22:299–307.

12. Chew MS, Poelaert J (2003) Accuracy and repeatability of pediatric cardiac output measurement using Doppler: 20-year review of the literature. Intens Care Med 29:1889–1894.

13. Paediatrics: WG on NHM in, Knirsch W, Kretschmar O, et al (2008) Comparison of cardiac output measurement using the CardioQPTM oesophageal Doppler with cardiac output measurement using thermodilution technique in children during heart catheterisation*. Anaesthesia 63:851–855.

14. Branski LK, Herndon DN, Byrd JF, et al (2011) Transpulmonary thermodilution for hemodynamic measurements in severely burned children. Crit Care 15:R118.

**7a. Transpulmonary dilution (TPD)**

**Short description of the parameter/method including reliability, normal or target values and estimated clinical usefulness**

The basic principle of transpulmonary dilution has been described many years ago[1]. It is based upon the injection of an indicator into a central venous vein. The indicator is detected downstream (arterially) through an indicator-sensitive device. The area under the indicator dilution curve represents the cardiac output. Because of variability in measurements secondary to cardiorespiratory interactions, the mean value of 2-3 measurements is generally taken. Although temperature was the first indicator used, several other indicators can be used, such as indocyanine green, lithium, or normal saline.

The transpulmonary thermodilution (TPTD) method is implemented in the PiCCO® (Pulsion, Germany) system and EV1000 (Edwards, USA) system. However, the latter device cannot be used in small children. The transpulmonary ultrasound dilution (TPUD) is incorporated in the COstatus device (Transonic®). The transpulmonary lithium dilution (TPLD) is incorporated in the LiDCO® device (LiDCO UK). This latter method is not usable in children < 40 kg. This document concerns only TPTD and TPUD, since they are applicable in small children.

TPTD and TPUD both measure several parameters including cardiac output, lung water and blood volumes. Additionally, the TPUD technology can detect and quantify shunts. Additional calculations can be done using cardiac output, blood volume and lung water. They indicate cardiac function and permeability of the pulmonary vessels.

Both TPTD and TPUD methods require central venous and arterial vascular access. The TPTD also requires a dedicated arterial catheter to be placed at the femoral artery level. With the smallest catheter being 3 Fr (7 cm), it can only be used in children > approximately 3.5 kg. TPUD requires any regular arterial and central venous catheters and is feasible in infants weighing >700 grams.

**Cardiac output**

The TPD method (both TPTD and TPUD) of cardiac output estimation is based upon the Stewart Hamilton calculation also used by pulmonary artery catheter measurements. It can be considered to be the clinical gold standard in children in accuracy and precision [2, 3]. This topic is covered in subgroup 4.

**Blood volume measurement**

Several blood volume categories can be measured depending on the technology used.

1. TPTD volume

Global End-Diastolic Volume (GEDV) is measured by the TPTD method and reflects blood volume between the injection and detection sites excluding the pulmonary blood volume [4]. It is indexed to the body surface area (GEDVI). Its values vary between 200-700 ml/m2, depending on age [5, 6]. GEDVI (indexed value) has shown to be related to the volume status, but it has not been shown to indicate fluid responsiveness in a reliable manner in children [5, 7].

2. TPUD volumes

**-**Total End-Diastolic Volume Index (TEDVI): this is the sum of the End-Diastolic Volumes of the atria and ventricles. It is indexed to body weight and the expected range is between 6-10ml/kg.

-Central Blood Volume Index (CBVI): this is the volume of blood between the injection site (central vein) and the recording site (artery). Blood in the heart, lungs, and large vessels contributes to the majority of CBV. It is indexed to body weight with a normal value between 18-22 ml/kg.

-Active Circulating Volume Index (ACVI): this is defined as the volume of blood in which the indicator mixes in one minute from the time of injection. ACVI is also indexed to body weight and the typical values are between 40- 60 ml/kg. In smaller children <5 kg it may reach up to 75 ml/kg.

All blood volumes have shown to reflect the blood volume status in experimental conditions [8, 9].

**Lung water**

TPD techniques can also estimate the amount of pulmonary edema (Extra-Vascular Lung Water or EVLW). Lung water is indexed to body weight (ml/kg) and its measurement is validated in adults and children against double indicator measurements but also in an experimental animal model against gold standard gravimetrics. In adults the precision seems adequate and it can track changes in a fast manner [6, 8, 10–12]. However, the clinical use of lung water estimation in children is not clear. In children, EVLW (by TPTD) did not correlate with a chest x-ray score [13]. Furthermore, EVLWI seems to be higher in younger children and is therefore not comparable to results in adults. Indexing to height instead of body weight seems more physiological and may provide an age-independent value [6].

There are no studies targeting hemodynamic therapy to lung water values. However, when using TPD methods an increasing value of lung water could indicate an increase in pulmonary edema that might warrant a more restrictive fluid therapy.

**Shunt detection**

TPUD may be used for qualitative and quantitative estimation of anatomical shunts based upon characteristics of the indicator dilution curves. Bi-directional shunts have demonstrated characteristic properties of both types of shunt, i.e. short appearance times and asymmetry of the downslope. In addition to the qualitative shunt detection, the software also provides a quantitative estimate of the pulmonary to systemic blood flow ratio (Qp/Qs). Moreover, it has been shown that cardiac output estimation is accurate in the presence of a substantial left-to-right transductal shunt volume. Saxena R et al reported TPUD to be an accurate (overall accuracy 86.1%) method for the detection of small anatomical shunts, both qualitatively and quantitatively [1, 14]. However, this is not yet proven useful in prospective clinical studies.

**Cardiac function**

The Cardiac Function Index (CFI) is calculated as the ratio between CI and GEDVI. It reflects the relation between cardiac output and blood volume. There are no data in children concerning this parameter. However, since cardiac output seems higher and GEDVI seems lower in young children it is to be expected that CFI values in younger children are higher compared to adults.

The Pulmonary Vascular Permeability Index (PVPI) reflects the relation between lung water and GEDVI. This parameter could indicate whether pulmonary edema is caused by hydrostatic pressure or by an increased permeability. There are no pediatric studies evaluating its clinical usefulness.

**References for TPD**

1. Zierler KL (1962) Theoretical Basis of Indicator-Dilution Methods For Measuring Flow and Volume. Circ Res 10:393–407. https://doi.org/10.1161/01.res.10.3.393

2. Sigurdsson TS, Aronsson A, Lindberg L (2019) Extracorporeal Arteriovenous Ultrasound Measurement of Cardiac Output in Small Children. Anesthesiology 130:712–718. https://doi.org/10.1097/aln.0000000000002582

3. Lemson J, Boode WP de, Hopman JCW, et al (2008) Validation of transpulmonary thermodilution cardiac output measurement in a pediatric animal model. Pediatr Crit Care Me 9:313–319. https://doi.org/10.1097/pcc.0b013e31816c6fa1

4. Proulx F, Lemson J, Choker G, Tibby SM (2011) Hemodynamic monitoring by transpulmonary thermodilution and pulse contour analysis in critically ill children. Pediatr Crit Care Me 12:459–466. https://doi.org/10.1097/pcc.0b013e3182070959

5. Oliva P de la, Menéndez-Suso JJ, Iglesias-Bouzas M, et al (2015) Cardiac Preload Responsiveness in Children With Cardiovascular Dysfunction or Dilated Cardiomyopathy. Pediatr Crit Care Me 16:45–53. https://doi.org/10.1097/pcc.0000000000000286

6. Nusmeier A, Cecchetti C, Blohm M, et al (2015) Near-Normal Values of Extravascular Lung Water in Children. Pediatr Crit Care Me 16:e28–e33. https://doi.org/10.1097/pcc.0000000000000312

7. Renner J, Broch O, Duetschke P, et al (2012) Prediction of fluid responsiveness in infants and neonates undergoing congenital heart surgery. Bja Br J Anaesth 108:108–115. https://doi.org/10.1093/bja/aer371

8. Vrancken SL, Nusmeier A, Hopman JC, et al (2016) Estimation of extravascular lung water using the transpulmonary ultrasound dilution (TPUD) method: a validation study in neonatal lambs. J Clin Monitor Comp 30:985–994. https://doi.org/10.1007/s10877-015-9803-7

9. Vigani A, Shih A, Queiroz P, et al (2012) Quantitative response of volumetric variables measured by a new ultrasound dilution method in a juvenile model of hemorrhagic shock and resuscitation. Resuscitation 83:1031–1037. https://doi.org/10.1016/j.resuscitation.2012.01.014

10. Lemson J, Backx AP, Oort AM van, et al (2009) Extravascular lung water measurement using transpulmonary thermodilution in children. Pediatr Crit Care Me 10:227–233. https://doi.org/10.1097/pcc.0b013e3181937227

11. Monnet X, Persichini R, Ktari M, et al (2011) Precision of the transpulmonary thermodilution measurements. Crit Care 15:1–6. https://doi.org/10.1186/cc10421

12. Dres M, Teboul J-L, Guerin L, et al (2014) Transpulmonary Thermodilution Enables to Detect Small Short-Term Changes in Extravascular Lung Water Induced by a Bronchoalveolar Lavage. Crit Care Med 42:1869–1873. https://doi.org/10.1097/ccm.0000000000000341

13. Lemson J, Hoeven JG van der, Die LE van, Hemelaar AE (2010) Extravascular lung water index measurement in critically ill children does not correlate with a chest x-ray score of pulmonary edema. Crit Care 14:R105. https://doi.org/10.1186/cc9054

14. Saxena R, Krivitski N, Peacock K, et al (2015) Accuracy of the transpulmonary ultrasound dilution method for detection of small anatomic shunts. J Clin Monitor Comp 29:407–414. <https://doi.org/10.1007/s10877-014-9618-y>.

**8. Pulmonary artery and left atrial pressure**

**Short description of the parameter/method**

Continuous measurement of pulmonary arterial pressure (PAP) is rarely performed in children [1]. Pulmonary artery catheter (PAC) represents the most accurate device for this purpose, allowing for continuous measurement of right atrial and ventricular pressures, PAP and when requested, pulmonary arterial occlusion pressure (wedge pressure). Left atrial pressure is eventually measured in cardiac patients as part of postoperative monitoring, particularly in procedures involving left-side structures,. The difference between pulmonary arterial and venous (or left atrial) pressures is known as Transpulmonary Gradient (TPG) and it is of diagnostic and prognostic importance in several cardiac conditions, such as patients with single ventricle circulation who are evaluated for total cavo-pulmonary connection or assessment of suitability for cardiac transplantation. The PAC also enables the measurement of cardiac output (see section cardiac output), CI and vascular resistances, and to take blood samples for mixed venous oxygen saturation (see section SmvO2 and ScvO2).

**Short overview of the reliability of the method**

Inserting a PAC is an invasive technique, difficult to place in children, particularly in those with abnormal cardiopulmonary anatomy. Intracardiac shunts, common in congenital heart disease, can render measurements invalid, limiting its applicability. Considerable experience is required for its insertion and the interpretation of curves. Consensus on the use of PAC in children was proposed in a statement of the Society of Critical Care Medicine in 1997 [2]. This document proposed four indications for PAC in children: a) pulmonary hypertension diagnosis and management b) shock refractory to fluid resuscitation and/or low-to-moderate dose of vasoactive agents; c) pediatric acute respiratory distress syndrome requiring high positive end expiratory pressure; d) multiple organ dysfunction on rare occasions. Currently though, PAC insertion is mostly restricted to cardiac catheterization laboratories for diagnostic, response to treatment and prognostication purposes [3, 4].

Over the last ten years, echocardiography has improved its ability to provide a reliable estimation of PAP [5]. Systolic PAP is considered equal to right ventricle systolic pressure in the absence of pulmonary valve stenosis or outflow tract obstruction. With cardiac ultrasound, right ventricle systolic pressure can be determined by the addition of right atrial pressure (commonly measured in any unstable critically-ill children) to the pressure gradient (Delta P) between the right chambers, calculated using the modified Bernoulli equation: Delta P= 4* (the peak velocity of the tricuspid valve regurgitation jet)2. To improve accuracy and limit angle dependency, the tricuspid regurgitation jet should be appreciated from multiple right ventricle views. However, in the setting of tricuspid regurgitation with laminar flow, the peak velocity does not evaluate correctly the gradient between the right ventricle and the right atrium because of early pressure equalization of right ventricle and right atrium, thus peak early diastolic and end-diastolic velocities obtained from pulmonary regurgitation can provide an estimation of the mean PAP and diastolic PAP.

Left atrial pressure is generally measured using left atrial catheters (LAC) or indirectly using the PAC with the measurement of the pulmonary occlusion wedge pressure[1]. LAC is placed at the time of cardiac surgery and represents the “gold standard” for left atrial pressure measurement. Alternative and less invasive techniques have been used in children to estimate left atrial pressure in unstable patients [6, 7]. The pulsed Doppler and tissue Doppler E/e’ ratio evaluate the relationship between diastolic transmitral flow velocity and myocardial relaxation velocity in the lateral aspect of the mitral annulus. Adult studies showed a good correlation between the wedge pressure measured with PAC and the echocardiographic analysis of the E/e’ ratio. However, data is still scanty in children. Currently, the E/e’ ratio is only able to estimate pulmonary wedge pressure; and may be useful predicting which subgroup of patients have elevated left atrial pressure, without the specificity that PAC and LAC can provide.

**Normal or target values**

Outside the neonatal period, normal PAP is estimated around one quarter to one third of the systemic arterial pressure. Standard diagnostic criteria for pulmonary hypertension include the presence of mean pulmonary artery pressure > 25 mmHg with pulmonary artery occlusion pressure <15 mmHg and pulmonary vascular resistance > 3 Wood units/m2.

Normal values for left atrial pressure and wedge pressure range between 6-12 mmHg.

**The estimated clinical usefulness**

PAP: Most commonly, echocardiography is the method of election due to its reproducibility and availability, especially in cardiac units. Some cardiac surgical patients will have a PA line inserted perioperatively and this will guide PA monitoring and/or treatment during the early postoperative state. Undesirable complications at time of removal include bleeding and tamponade, due to the location of the line (main pulmonary artery). PA lines are therefore preferably removed at the time of sternotomy closure or with mediastinal/pleural drains in situ.

LAP: Direct measurement of LAP with invasive lines provides useful information of LA filling pressure, mitral valve performance, rhythm and distensibility of LV in an array of cardiac conditions. Pulmonary wedge pressure provides information about post-capillary pressure at the time of measurement when a PAC is used and it is currently not a common occurrence in the PICU environment, rather more in catheter laboratory. Echocardiography can also provide, as seen above, valuable information about LA anatomy, function and grossly discriminate those patients likely to have high LAP. This is particularly useful when concerns exist about LA hypertension and there are no invasive measurement alternatives, except cardiac catheterization.

**References for pulmonary artery pressure**

1. Perkin RM, Anas N (2011) Pulmonary artery catheters. Pediatr Crit Care Me 12:S12–S20. https://doi.org/10.1097/pcc.0b013e318220f079

2. (1997) Pulmonary Artery Catheter Consensus Conference: Consensus Statement. Crit Care Med 25:910. https://doi.org/10.1097/00003246-199706000-00006

3. Mullen MP (2010) Diagnostic strategies for acute presentation of pulmonary hypertension in children: Particular focus on use of echocardiography, cardiac catheterization, magnetic resonance imaging, chest computed tomography, and lung biopsy. Pediatr Crit Care Me 11:S23–S26. https://doi.org/10.1097/pcc.0b013e3181c7683a

4. Matsuura H (2017) Cardiac catheterization in children with pulmonary arterial hypertension. Pediatr Int 59:3–9. https://doi.org/10.1111/ped.13161

5. Moceri P, Baudouy D, Chiche O, et al (2014) Imaging in pulmonary hypertension: Focus on the role of echocardiography. Arch Cardiovasc Dis 107:261–271. https://doi.org/10.1016/j.acvd.2014.02.005

6. Figueras-Coll M, Sanchez-de-Toledo J, Gran F, et al (2015) Echocardiography in the Assessment of Left Atrial Pressure After Pediatric Heart Surgery. World J Pediatric Congenit Hear Surg 6:438–442. https://doi.org/10.1177/2150135115589999

7. Goldberg DJ, Quartermain MD, Glatz AC, et al (2011) Doppler tissue imaging in children following cardiac transplantation: A comparison to catheter derived hemodynamics. Pediatr Transplant 15:488–494. <https://doi.org/10.1111/j.1399-3046.2011.01503.x>

**9. Fluid responsiveness**

**Short description of the parameter/method**

Volume resuscitation of hemodynamically unstable patients is one of the most important therapeutic options available. Unfortunately though, excessive fluid administration may impair tissue perfusion even further by promoting edema and third-space accumulation [1,2]. Endothelial dysfunction and capillary leak are frequently present in shock states and contribute to undesired fluid shifts. Fluid overload signs (tissue edema, ascites, pleural effusions, pulmonary edema, and other third space fluid accumulation) can often coexist with hypovolemia.

It would be ideal to “predict” when fluid is going to be beneficial (ultimately, when it is going to improve perfusion) by any possible means [1-6]. Static measures (like the so-called ”filling” pressures) are frequently used to manage fluid therapy to a certain Central Venous Pressure (CVP) target, even when their predicting value has been showed to be close to 0.5 (no higher than chance) [7]. On the other hand, dynamic measures have been proposed, validated and are being increasingly adopted. These measures and tests for fluid responsiveness have been reviewed in adults and pediatric patients recently [6-9]. Briefly, breathing-induced changes over time in cardiac output indicators (stroke volume variation (SVV), systolic pressure variation (SPV), pulse pressure variation (PPV)) amongst others can be tracked by different means such as transpulmonary thermodilution, echocardiography, pulse contour analysis, blood pressure analysis, plethysmography, transesophageal Doppler and other technologies when circulation is challenged with fluid by positional changes (passive leg raising, liver compression in small children and infants) or fluid administration. Several cut-offs have been described to distinguish volume responders from those who are not and therefore will not benefit from fluid administration. Physiologically, the position of each individual’s ventricular function on the Frank-Starling curve will determine what will happen to the cardiac output after fluid is administered. The effect of positive pressure ventilation on right and in turn left cardiac output -globally referred to as cardiopulmonary interactions- is an essential part of these measures, and also part of their limitations [1-9]. Furthermore, pediatric studies are far more scarce and present unique challenges [5,8-11]. Differences in tissue compliance and the small volumes used in children (both to expand or to track changes) may explain why most of the dynamic tests validated in adults are not reliable in children.

**Short overview of the reliability of the method if applicable**

In adult patients PPV has rendered the highest values of discriminative cut-off for fluid responsiveness, closely followed by SVV and SPV [3,6]. Limitations to its use include spontaneous breathing, small tidal volumes and high frequency ventilation, open chest, high intra-abdominal pressure and cardiac arrhythmias. End-expiratory occlusion test can be performed in spontaneously breathing and / or arrhythmic patients as long as triggering efforts during the pause (15 seconds) do not interfere with the measurement. They also work better with continuous cardiac output monitoring rather than blood pressure (BP). This test has been reported in adults so far [7]. Two recent systematic reviews of pediatric studies [8,9] have not found the same good predictive value for blood-pressure and cardiac output derived dynamic variables.

Respiratory variation in aortic blood flow peak velocity (ΔVPeak) was the best predictor of fluid responsiveness in children, although a cut-off value could not be identified. In the systematic review and meta-analysis by Desgranges *et al* the pooled sensitivity and specificity of ΔVPeak for the overall population was 92.0% (95% CI 84.1–96.7) and 85.5% (95% CI 75.6–92.5) respectively. Cutoff values for ΔVPeak to predict fluid responsiveness varied across studies, ranging from 7% to 20%. The area under the SROC curve of ΔVPeak to predict fluid responsiveness was 0.94. Another potential disadvantage of ΔVPeak is the fact that it uses pulsed Doppler echocardiography and therefore can be subject to inter and intraobserver variations. Furthermore, there is a potential coupling between ΔVPeak and its efficacy-indicators, namely stroke volume (SV), stroke volume index (SVI) and aortic velocity time integral, because they all are obtained with pulsed Doppler [9].

Autotransfusion of fluid (by shifting blood from venous pools into the right ventricle (RV), thereby increasing preload) is a well-explored concept in adult literature and has rendered good results in determining fluid responsiveness [3,7]. In children there is a paucity of studies and lack of homogeneity. Lukito and coworkers [10]. performed a passive leg raising manoeuvre in 40 pediatric patients and found that 50% were responders by increasing their cardiac index >10%. These figures are in agreement with the usual proportion of adult responders to passive leg raising. Lu et al [11]. observed a wide variation of CI after PLR in children under 5 years-old, but better results in older children, with a sensitivity of 100% and specificity of 25% (PPV 40%, NPV 100%). Lee *et* al [12] studied the effect of liver compression in a group of 30 children of less than 5 years of age post-cardiac surgery. They found that fluid responsiveness was predicted by changes in diastolic blood pressure (ΔDAP) and ΔVPeak with cut-off points of 5% and 12% respectively. Changes in systolic blood pressure (ΔSAP), SPV and CVP were not discriminative. Static variables have not shown value for fluid response prediction in children as well as in adults. In both studies, echocardiography was used to assess response.

Respiratory variation in IVC diameter measured by ultrasound has limited ability to predict fluid responsiveness in children, according to the last meta-analysis published [13]. It seems clear that fluids could be safely administrated if the variability is greater than 18%, and withheld if it is lower than 3%, but most of the patients would present with intermediate values, difficult to interpret [14].

No studies regarding the ability to predict FR of the mini-fluid challenge in children have been reported. There are no studies in children involving ECLS or ARDS and fluid response.

**Short overview concerning the estimated clinical usefulness**

Despite limitations in currently available studies, assessment of fluid responsiveness by dynamic variables should be an important part of pediatric intensive care and anesthesia. Moreover, there is a need to move away from CVP targets and excessive fluid challenge triggered by blood pressure changes as an automatic, rather than a well thought-through therapeutic act. Unfortunately, these two misconceptions are still too frequent in daily practice.

Obviously, more studies are needed, and better tools required in order to create protocols such as those currently available for adult patients. Physiological differences between children and adults are, at least partly, the reason why successful measurements such as PPV and SPV are not getting equal results when tested in pediatric populations. In addition, children with congenital heart disease and specifically extensive aortic surgery may be particularly difficult to assess in terms of reliable quantification of cardiac output, arterial elastance and ultimately hemodynamic status due to technical limitations of current monitoring options. Perhaps better patient selection and multicenter approach to increase patient recruitment and statistical significance are the way forward.

Awareness of timing, adequacy and amount of fluid resuscitation should be the result of a multi-layered thinking process. Not forgetting that even in “fluid-responders” there is the ultimate question of actual improvement of microcirculatory and tissue perfusion, a field yet to be fully explored. But despite these limitations, it is conceivable that when well executed, patients will undoubtedly benefit from fluid responsiveness evaluation. Especially those who are more severely ill.

In conclusion, there is no clear and proven method to predict fluid responsiveness in children. Static measures, mostly CVP are not appropriate to test fluid responsiveness. However, a clear increase in CVP after fluid loading indicates fluid overload and suggests to deliver no further fluids.

The literature indicates that ΔVPeak is the most reliable indicator of fluid responsiveness. Nevertheless, this holds only true in ventilated children that fulfil various criteria. Other methods (like PLR and liver pressure) are not thoroughly tested in children of all ages. Physiologically the most optimal method today would be to give a small bolus of fluid in a very short period and observer a change in cardiac output. This has only been tested in adults so far.

For clinical purposes one must bear in mind that identifying a patient as a fluid responder does not mean the patient is in need for fluid therapy. Therefore, no fluid bolus should be given without 1) a strong suggestion, based upon the patient’s clinical situation, physical exam, and various perfusion indicators, of a too low cardiac output (or oxygen delivery) caused by hypovolemia and 2) an attempt to predict fluid responsiveness, except in obvious resuscitation circumstances.

**Proposed algorithm for fluid assessment, for all patient subtypes.**

1. Observe all available information with regard to the hemodynamic situation for suggestion of a low cardiac output or oxygen delivery caused by hypovolemia.
2. Consider the clinical situation, disease period (early vs. late resuscitation) and cumulative fluid balance.
3. Consider what available perfusion indicators suggest.
4. Attempt to predict fluid responsiveness using available equipment with regard to all limitations.
5. Give a small fluid bolus in a short time period and observe effect.
6. Consider other factors and therapies (inflammation, vasomotor modulation, inotropic support, sedation titration) that can improve the circulatory status without repeated fluid administration.

**References for fluid resuscitation and responsiveness**

1. Sethi, SK, et al (2018) Fluid Overload and Renal Angina Index at Admission Are Associated With Worse Outcomes in Critically Ill Children. Front Pediatr. 6:118.
2. Selewski D, et al (2017) The Impact of Fluid Overload on Outcomes in Children Treated With Extracorporeal Membrane Oxygenation: A Multicenter Retrospective Cohort Study. Pediatr Crit Care Med. 18:1126–1135.
3. Monnet et al (2013). Assessment of volume responsiveness during mechanical ventilation: recent advances. Critical Care, 17:217.
4. Pranksunas et al (2013). Microcirculatory blood flow as a tool to select ICU patients eligible for fluid therapy. Intensive Care Med 39:612–619.
5. Proulx et al (2011) Hemodynamic monitoring by transpulmonary thermodilution and pulse contour analysis in critically ill children. Pediatr Crit Care Med; 12:459 –466.
6. Marik et al (2009) Dynamic changes in arterial waveform derived variables and fluid responsiveness in mechanically ventilated patients: A systematic review of the literature. Crit Care Med; 37:1-6.
7. Monnet et al (2016) Passive leg raising for predicting fluid responsiveness: a systematic review and meta-analysis. Intensive Care Med 42:1935–1947
8. Gan et al (2013) Predicting fluid responsiveness in children: A systematic review. Anesth Analg; 117:1380–92.
9. Desgranges et al (2016) Respiratory variation in aortic blood flow peak velocity to predict fluid responsiveness in mechanically ventilated children: a systematic review and meta-analysis. Paediatr Anaesth; 26:37-47.
10. Lukito et al (2012) The role of passive leg raising to predict fluid responsiveness in pediatric intensive care unit patients. Pediatr Crit Care Med; 13:e155–e160.
11. Lu GP, Yan G, Chen Y, Lu ZJ, Zhang LE, Kissoon N (2015) The passive leg raise test to predict fluid responsiveness in children--preliminary observations. Indian J Pediatr; 82:5-12.
12. Lee et al (2017) Prediction of fluid responsiveness based on liver compression-induced blood pressure changes in children after cardiac surgery. Minerva Anestesiol; 83:939-46.
13. Long E, Oakley E, Duke T, Babl FE; Paediatric Research in Emergency Departments International Collaborative (PREDICT) (2017) Does respiratory variation in inferior vena cava diameter predict fluid responsiveness: a systematic review and meta-analysis. Shock; 47: 550-9.
14. Millington SJ (2019). Ultrasound assessment of the inferior vena cava for fluid responsiveness: easy, fun, but unlikely to be helpful. Can J Anaesth; 66:633-638.

**10. Near-infrared spectroscopy measurement**

**Short description of the parameter/method**

Near-infrared spectroscopy (NIRS) is a non-invasive, bedside technique to estimate capillary-venous hemoglobin saturation (rSO2). A near infrared light between 700-900 nm allows light to pass through skin or bone into underlying tissue (1,2). The values obtained with NIRS represent primarily the oxygenation status of the chromophores of the venous compartment, representing 75% of cerebral vascular bed, with respect to arterial and capillary bed, representing 20% and 5%, respectively (3).

**Short overview of the reliability of the method**

The most common application of NIRS is the assessment of cerebral rSO2 (cRSO2) using sensors placed on the patient's forehead (3). cRSO2 derives mostly from the balance between oxygen delivery and utilization in the gray matter in the frontal region. It is recommended that the cerebral probe be placed on the right or left side of the forehead and away from nevi, sinus cavities, the superior sagittal sinus, subdural or epidural hematomas, or other anomalies such as arteriovenous malformations. Because of the small surface area available on the forehead, the midline position has been used successfully for monitoring cRSO2 in neonates and small infants (4,5).

In neonates and infants, NIRS-measured RSO2 of deeper organs like kidneys (rRSO2) or intestines (sRSO2) is feasible due to their superficial location (6), although their use in clinical practice remains questionable because of non-specificity. The use of transcutaneous NIRS is safe and no adverse effects (such as skin burns) have been reported even with prolonged use (6).

In summary, use of NIRS for monitoring cRSO2 is fairly reliable. However, there remains lack of standards on its use for different ages, from preterm infants to children.

**Normal or target values**

Standardized values in healthy preterm and term infants have been recently published: mean baseline cerebral rSO2 >70% has been reported in healthy infants and children and those with acyanotic heart disease. These values are similar to the adult population. In comparison, patients with cyanotic heart disease have a mean cerebral rSO_2_ between 46-57% (7-11).

Clinical data in children and adults support the hypothesis that cerebral rSO2 under 40-50% or a change in baseline of more than 20%, is associated with hypoxic-ischemic neural injury (12). In general, a decrease in rSO2 is reflective of an increase in oxygen extraction and debt as a result of increased metabolism, decreased perfusion, and/or stagnant perfusion. Thus, monitoring for major patient-specific trends rather than an absolute number (especially those trends that often appear before any other clinical signs) become important in any given clinical setting (4).

**Short overview concerning the estimated clinical usefulness**

NIRS has been integrated in the evaluation of patients in the ICU during cardiac surgery showing a correlation with central venous saturation measured in the superior vena cava and an early detection of patient at risk of low cardiac output (13). The significant correlation has been described also in the postoperative period in a cohort of neonates with cyanotic and acyanotic cardiac heart diseases undergoing cardiac surgery (14). Hansen et al. (15) showed a relation between low rScO2 (and SvO2) in the initial postoperative period and postoperative complications in patients undergoing superior cavo-pulmonary anastomosis. In a study by Dabal et al. (16), it appears that renal NIRS and inferior vena cava desaturations precede rScO2 changes in the prediction of serious cardiovascular adverse events in patients after stage 1 Norwood palliation. However, the evidence for the routine application of NIRS outside the peri-operative period for cardiac surgery remains poor (14).

Similarly, application of NIRS has been studied in emergency situations needing chest compression or post-cardiac arrest, traumatic brain injury and hypoxic ischemic encephalopathy (HIE) (15-21). Some centers are using NIRS in HIE, preterm infants or for other indications as above.

In summary, currently in most neonatal and pediatric intensive care units routine use of NIRS is primarily useful in the perioperative course of cardiac surgery.

**References for NIRS**

1. Ghanayem N., Wenovsky G, Hoffman G. Near-infared spectroscopy as hemodynamic monitor in critical illness Pediatr Crit Care med 2011; 12(4) :S27-S32.
2. Sood BG., McLaughlin K., Cortez J. Near-infrared spectroscopy: applications in neonates Semin Fetal Neonatal Med 2015;20(3):164-72.
3. Drayna P., Abramo T, Estrada C. Near-infrared spectroscopy in the critical setting Pediatric Emergency Care 2011;27(5):432-442.
4. Garner RS, Burchfield D. Treatment of presumed hypotension in very low birthweight neonates: effect on regional cerebral oxygenation. Arch Dis Child Fetal Neonatal Ed. 2010;95(3):F213-9.
5. Sood BG, Cortez J , McLaughlin KL et al. Near infrared spectroscopy as a biomarker for necrotizing enterocolitis following red blood cell trasfunsion. J Near InfraRed Spectrosc 2014;22(6):375-88.
6. Schat TE, Van der Laan ME, Schurink M et al Abdominal near-infrared spectroscopy in preterm infants: a comparison of splanchnic oxygen saturation measurements at two abdominal locations Early Hum Dev 2014;90(7)371-5.
7. Tina LG, Frigiola A, Abella R et al Near infrared spectroscopy in healthy preterm and term newborns: correlation with gestational age and standard monitoring parameters. Curr Neurovasc Res 2009; 6(3):148-154.
8. Bernal NP, Hoffman GM, Ghanayem NS et al Near-infrared spectroscopy in normal newborns J pediatric Surg 2010;45:1306-1310.
9. Johnson BA, Hoffmann GM, Tweddell JS et al Near-infarred spectroscopy in neonates before palliation of hypoplastic left heart syndrome Ann Thorac Surg 2009;87:571-577.
10. Kurth CD, Steven JL, Montenegro LM et al Cerebral oxygen saturation before congenital heart surgery . Ann Thor Surg 2011;72:187-192.
11. Fenton KN Freeman K, Glogowski K et al The significance of baseline cerebral oxygen saturation in children undergoing congenital heart surgery Am J Surg 2005;190:260-263.
12. Hoffman GM, Ghanayem NS, Tweddell JS. Noninvasive assessment of cardiac output. Semin Thorac Cardiovasc Surg Pediatr Card Surg Annu. 2005:12Y21.
13. Ranucci M, Isgro G, Dela Torre T, et al. Near-infrared spectroscopy correlates with continuous superior vena cava oxygen saturation in pediatric cardiac surgery patients. Paediatr Anaesth 2008; 18:1163–1169.
14. Zulueta JL, Vida VL, Perisinotto E, et al. Role of intraoperative regional oxygen saturation using near infrared spectroscopy in the prediction of low output syndrome after pediatric heart surgery. J Card Surg 2013; 28:446-452.
15. Bronicki RA, Herrera M, Mink R, et al. Hemodynamics and cerebral oxygenation following repair of tetralogy of Fallot: the effects of converting from positive pressure ventilation to spontaneous breathing. Congenit Heart Dis 2010; 5:416-421.
16. Hansen JH, Schlangen J, Armbrust S, et al. Monitoring of regional tissue oxygenation with near-infrared spectroscopy during the early postoperative course after superior cavopulmonary anastomosis. Eur J Cardiothorac Surg 2013; 43:e37–e43.
17. Dabal RJ, Rhodes LA, Borasino S, et al. Inferior vena cava oxygen saturation monitoring after the Norwood procedure. Ann Thorac Surg 2013; 95:2114– 2120.
18. Mullner M, Sterz F, Binder M, et al. Near infrared spectroscopy during and after cardiac arrest preliminary results. Clin Intensive Care. 1995;6(3):107Y111.
19. Gomez-Pesquera E, Poves-Alvarez R, Martinez Rafael et al Cerebral oxygen saturation and negative postoperative behavioral changes in pediatric surgery: a prospective observational study J Pediatr 2019;208:207-13.
20. Wagner BP, Pfenninger J. Dynamic cerebral autoregulatory response to blood pressure rise measured by near-infrared spectroscopy and intracranial pressure. Crit Care Med. 2002;30(9):2014Y2021.
21. Adelson PD, Nemoto E, Colak A, et al. The use of near infrared spectroscopy (NIRS) in children after traumatic brain injury: a preliminary report. Acta Neurochir Suppl. 1998;71: 250Y254.

**11. Microcirculation and venous to arterial CO2 difference**

**Short description of the parameter/method**

Microcirculation is the set of small size blood vessels interspersed between the arterial and the venous circulations, where the exchange of oxygen, nutrients and waste substances between blood and tissues takes place. Microcirculation regulation is very complex with multiple factors and mechanisms interacting both at local and global levels.

Several methods for the evaluation the microcirculation have been proposed; namely clinical examination (capillary refill or gradient from central to peripheral temperature), biochemical parameters (lactate, pH, hyaluronan), laser doppler flow, near-infrared spectroscopy (NIRS), PaO2 and tissue metabolites measured by electrodes or microdialysis and videomicroscopic techniques. In this section we are only going to analyze the latter.

Videomicroscopy allows a live-view of the network of capillaries, arterioles and venules of the microvascular system. Basically, every capillary vascular system can be used to study. The only requisite is that the camera system must be positioned 1-2 mm away from the capillaries. Therefore, clinically the most commonly accessed area is the sublingual or buccal mucosa. In neonates inner arm skin has also been used [1,2].

Several techniques of hand-held videomicroscopy are available:

1. Orthogonal Polarization Spectral (OPS): illuminates the tissue through a spectral polarizing filter that only allows light within the wavelength of the absorption spectrum of hemoglobin. Blood vessels are traced by the cells with hemoglobin that circulate inside them.

2. Sidestream Dark Field (SDF): Beams of light that impinge on the point to be evaluated from a ring of concentric LED lights.

3. Incident Dark Field (IDF): allows to obtain images with a greater field of vision and higher resolution.

Central venous to arterial CO2 difference (AVCO2) measures the circulatory clearance of tissue CO2. In adult studies AVCO2 is correlated with cardiac output [3,4]. In low cardiac output and/or severe hypoxia tissue CO2 production is increased because hydrogen ions are buffered by bicarbonate. So venous CO2 increases and therefore central venous to arterial CO2 difference (AVCO2). For this reason, central venous to arterial CO2 difference could be a marker of impaired tissue perfusion [3,4]

**Short overview of the reliability of the method if applicable**

All three videomicroscopy methods can be used in children. There is an established systematic approach for quality measurement, evaluation and analysis of images [1]. The practical problem lies in the way video images are converted into numbers. Several software solutions exist but often also manual scoring is necessary or used. Therefore, results with different cameras and/or different software are difficult to compare.

Although other areas of the body have been used in the newborn [2], the sublingual area and buccal mucosa are the most used in adults and older children [1, 5-10].

Microcirculation assessment in pediatrics is feasible and good quality images can be obtained across a variety of age range and severity of clinical conditions. No complications have been described with the use of these techniques.

Videomicroscopy can identify microcirculatory alterations during shock, postoperative states, mechanical support and other critical care conditions as well as its response to treatment. Frequently reported are decreased density of perfused vessels (PVD) and microvascular flow index (MFI) alongside increased heterogeneity index (HI).

Nevertheless, several limitations limit its application in everyday clinical practice:

1. Videomicrocopy is not available in most of Pediatric Intensive Care Units.

2. Acquisition of images is very complicated in children who are not deeply sedated, which limits the generalization of its use [7]. Neonatal reports provided image acquisition on inner arm skin or auricular pavilion of non-sedated patients, but this location cannot be used in children.

3. It is not possible to perform a fast and automatic analysis to guide clinical decision in critically-ill children, although new software is being developed for that purpose.

4. Most studies have been performed in adults and neonates [2], with fewer studies in critically-ill children [5-10].

5. No clinical studies that have clearly defined prognostic value or treatment guidance possibilities for microcirculatory alterations in critically-ill children have been published.

Therefore, videomicrocopy is currently a technique used in research rather than a more widely available monitoring tool. The assessment of microcirculation has been described in PICU patients in different critical states such as cardiac arrest, shock of all etiologies, cardiac surgery, cardiac failure or mechanical support [7].

**Establish, when possible, normal or target values**

**Microcirculatory variables:**

- Variables related to the diffusion distance of oxygen:  FCD (Functional Capillary Density), TVD (Total Vessel Density), De Backer score, PVD (Perfused Vessel Density)
- Semi-quantitate assessment of average red blood cell (RBC) speed per quadrant:  MFI (Microvascular Flow Index). Scoring as normal (3), sluggish (2), intermittent (1) and absent (0) flow.
- Heterogeneity Index: using MFI or PPV. This is ratio of (fastest-slowest value)/ mean. A high heterogenous blood flow pattern suggests distributive shock pathophysiology.

No studies have defined the normal values ​​of microcirculation in children outside the neonatal period. These studies showed that vascular density seems to decrease with age [11].

So far, no studies that have defined target values of microcirculatory parameters in critically-ill children.

Normal values of central venous to arterial CO2 difference in children are not stablished. Most adult studies used a cutoff value of 6 mmHg [3, 4].

**Short overview concerning the estimated clinical usefulness**

Microcirculation is a crucial part of the vascular system directly involved in the supply of nutrients and oxygen, elimination of metabolic products to and from tissues. Microcirculatory alterations are seen in shock and critical illness and are related to prognosis in adults and some small pediatric studies [5,12].

Moreover, these microcirculatory abnormalities have shown lack of correlation with macrohemodynamic parameters both in adult and pediatric studies. Resuscitation guided by such parameters may be inadequate at the microvascular level, with potential outcome implications. Therefore, direct visualization of the microcirculation is important in critical care patients, including children. These devices are relatively non-invasive and no complications have been reported, even in anticoagulated patients on mechanical support. However, videomicroscopy is not widely available in PICUs. Children must be sedated for good image acquisition and analysis is still time-consuming. These caveats limit their widespread clinical applicability.

There is a need to continue developing this technology in critically-ill neonates and children. A specific pediatric consensus recommendation to study the microcirculation is lacking. Studies in non-critically-ill children under anesthesia should be conducted to develop normal values in children and neonates. More studies are required to evaluate microcirculation in unstable patients with hemodynamic compromise and to follow its response to therapy. Cardiac subgroups (single ventricle, cyanotic congenital heart disease, heart failure, mechanical support) warrant specific studies. The type of flow (continuous vs pulsatile) when children are supported with ECLS (VA) would be considered. The evaluation of microcirculation by videomicroscopy in critically-ill neonates has the same indications than in critically-ill children and is easier to measure in newborns, both in mucosa and skin locations.

Some studies in adult with sepsis shock and after surgery found that AVCO2 could be a prognostic marker [3, 4]. Still, there are very few studies that have analyzed the clinical usefulness of AVCO2 in critically-ill children , all of them performed after cardiac surgery, and with inconsistent results [13-15].

**References for microcirculation**

1. Ince C,   Boerma EC , Cecconi M, et al. (2018) Second consensus on the assessment of  sublingual  microcirculation  in critically ill patients: results from a task force of the European Society of Intensive Care Medicine.  Intensive care Medicine 44: 281- 299.
2. Genzel-Boroviczeny O, Strotgen J, Harris AG, et al. (2002) Orthogonal polarization spectral imaging (OPS): a novel method to measure the microcirculation in term and preterm infants transcutaneously. Pediatr Res. 51:386–91.
3. [Mallat J](https://www.ncbi.nlm.nih.gov/pubmed/?term=Mallat%20J%5BAuthor%5D&cauthor=true&cauthor_uid=26855893), [Lemyze M](https://www.ncbi.nlm.nih.gov/pubmed/?term=Lemyze%20M%5BAuthor%5D&cauthor=true&cauthor_uid=26855893), [Tronchon L](https://www.ncbi.nlm.nih.gov/pubmed/?term=Tronchon%20L%5BAuthor%5D&cauthor=true&cauthor_uid=26855893),  et al. (2016) Use of venous-to-arterial carbon dioxide tension difference to guide resuscitation therapy in septic shock. [World J Crit Care Med.](https://www.ncbi.nlm.nih.gov/pubmed/?term=Use+of+venous-to-arterial+carbon+dioxide+tension+difference+to+guide+resuscitation+therapy+in+septic+shock)  5:47-56.
4. [He H](https://www.ncbi.nlm.nih.gov/pubmed/?term=He%20H%5BAuthor%5D&cauthor=true&cauthor_uid=28492385), [Long Y](https://www.ncbi.nlm.nih.gov/pubmed/?term=Long%20Y%5BAuthor%5D&cauthor=true&cauthor_uid=28492385), [Liu D](https://www.ncbi.nlm.nih.gov/pubmed/?term=Liu%20D%5BAuthor%5D&cauthor=true&cauthor_uid=28492385),  et al (2017) The Prognostic Value of Central Venous-to-Arterial CO2 Difference/Arterial-Central Venous O2 Difference Ratio in Septic Shock Patients with Central Venous O2 Saturation ≥80. Shock 48:551-557.
5. Top APC, Ince C, de Meij N et al, (2011) Persistent low microcirculatory vessel density in nonsurvivors of sepsis in pediatric intensive care. Crit Care Med 2011; 39:8 –13.
6. Top APC, Ince C, Schouwenberg PHM, et al (2011) Inhaled nitric oxide improves systemic microcirculation in infants with hypoxemic respiratory failure. Pediatr Crit Care Med ; 12:e271– e274.
7. González R, López J, Urbano J, et al (2017) Evaluation of sublingual microcirculation in a pediatric intensive care unit: prospective observational study about its feasibility and utility. BMC Pediatrics 17:75
8. Scolleta, S. Marianello D, Isgrò G et al. (2016) Microcirculatory changes in children undergoing cardiac surgery: a prospective observational study B J A 117: 206–213
9. Schinagl M, Mormanova ZH, Puchwein-Schwepcke A (2016) The effect of red blood cell transfusion on the microcirculation of anemic children. Eur J Pediatr 175:793–798.
10. Top A, van Dijk M, van Velzen JE, et al (2011) Functional Capillary Density decreases after the first week of life in term neonates. Neonatology 2011;99:73–77.
11. Paize F, Sarginson R, Makwana N, et al (2012) Changes in the sublingual microcirculation and endothelial adhesion molecules during the course of severe meningococcal disease treated in the pediatric intensive care unit. Intensive Care Med 38:863–871.
12. Rhodes LA, Erwin WC, Borasino S, et al. Kuiper JW. Tibboel D, Ince C (2017). Central venous to arterial CO2 difference after cardiac surgery in infants and neonates. Pediatr Crit care Med 18:228-233
13. Akamatsu T, Inata Y, Tachibana K, et al (2017) Elevated central venous to arterial CO2 difference is not associated with poor clinical outcomes after cardiac surgery with cardiopulmonary bypass in children. Pediatric Crit care Med 2017;18:859-862
14. Furgan M, Hashmatt F, Amanullah M, et al (2009) Venoarterial PCO2 diffrence: a marker of postoperative cardiac output in children with congenital heart disease J Coll Physicians Surg Park 19:640-643.
